# Pre-analytical Best Practices for RNA Sequencing from Small Biopsies and Cytologic Specimens

**DOI:** 10.1007/s40291-026-00837-6

**Published:** 2026-03-14

**Authors:** Gloria Hopkins Sura, Kelly B. Engel, Sarah R. Greytak, Sandra M. Gaston, Kelsey Dillehay McKillip, Abhilasha Rao, Ping Guan, W. Fraser Symmans, Rachael Clark, Maria Arcila, Sayak Ghatak, Karol Bomsztyk, Lokesh Agrawal

**Affiliations:** 1 Division of Pathology and Laboratory Medicine, The University of Texas MD Anderson Cancer Center, Houston, TX USA; 2 GAP Solutions, Inc., Herndon, VI USA; 3 Kelly Government Solutions, Rockville, MD USA; 4 Desai Sethi Urology Institute, University of Miami Miller School of Medicine, Miami, FL USA; 5 Department of Pathology and Laboratory Medicine, University of Cincinnati Health Precision Medicine Lab, University of Cincinnati, Cincinnati, OH USA; 6 Biorepositories and Biospecimen Research Branch (BBRB), Division of Cancer Treatment and Diagnosis, Cancer Diagnosis Program, National Cancer Institute, Bethesda, MD USA; 7 Division of Pathology and Laboratory Medicine, The University of Texas MD Anderson Cancer Center, Houston, TX USA; 8 Department of Dermatology, Brigham and Women’s Hospital, Harvard Medical School, Boston, MA USA; 9 Department of Pathology and Laboratory Medicine, Memorial Sloan Kettering Cancer Center, New York, NY USA; 10 Frederick National Laboratory for Cancer Research, Frederick, MD USA; 11 UW Medicine South Lake Union, University of Washington, Seattle, WA USA; 12 Institute for Stem Cell and Regenerative Medicine, University of Washington, Seattle, WA USA

## Abstract

RNA sequencing is becoming increasingly common in precision oncology, in both clinical and research settings, for transcriptome profiling, gene-fusion detection, and biomarker discovery. RNA-sequencing analysis of specimens obtained via minimally invasive procedures such as small biopsy, fine needle aspiration, and exfoliation offers a powerful method for analyzing gene expression patterns and detecting RNA-level changes associated with cancers that are either difficult to collect or require longitudinal sampling. However, pre-analytical factors (e.g., details of specimen collection, processing, and storage workflow) influence not only RNA-sequencing success rates but also the quality and accuracy of sequencing results, which may affect patient care and research progress. Minimally invasive specimens are associated with a unique set of pre-analytical challenges owing to their small size, limited RNA yield, and distinct workflows. To address the need for evidence-based guidance, this review by a working group of National Cancer Institute grantees and intramural and extramural researchers identifies pre-analytical best practices for minimally invasive specimens destined for RNA-sequencing analysis, based on the available literature and their collective experience. Strategies for assessing specimen adequacy and RNA quality, maximizing tumor content, and minimizing specimen loss and RNA degradation due to pre-analytical handling are specified for small tissue and cytology specimens. By integrating current evidence and institutional insights, this review provides a practical framework for enhancing RNA-sequencing reliability and reproducibility in both clinical and research workflows.

## Plain Language Summary

RNA, or ribonucleic acid, carries the instructions that cells use to function and grow. RNA sequencing is a laboratory method that allows scientists to see which genes are active or altered. In cancer, this information helps explain how tumors behave, change over time, and respond to treatment, and it can support diagnosis and treatment planning. RNA sequencing is especially valuable when only small samples are available, such as needle biopsies, fine-needle aspirations, or cells released into body fluids. These minimally invasive samples are often the only practical option when tumors are difficult to reach or when repeat testing is needed. However, working with small samples is challenging because the amount of RNA is limited and easily damaged. Problems during collection, storage, or processing can reduce RNA quality and lead to unreliable results. To address these challenges, experts supported by the National Cancer Institute reviewed both published studies and real-world laboratory experience to develop practical guidance on how to collect, handle, and preserve small samples for RNA sequencing. Following these best practices improves the reliability of testing and helps ensure that meaningful information can be obtained even from very limited material.

**Graphical Abstract:**

## Graphical abstract

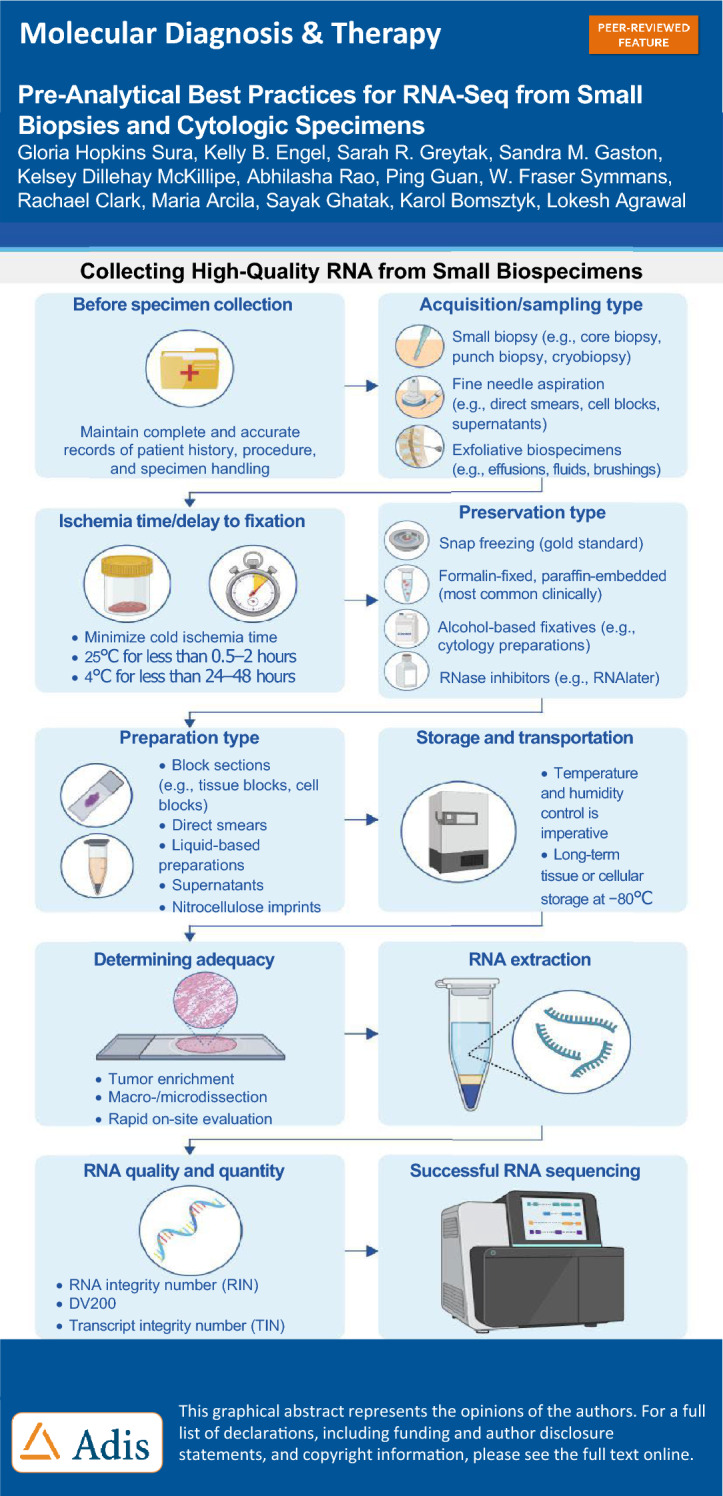

## Key Points

**Table:** 

RNA quality depends heavily on how the sample is handled before testing. For small biopsies and cytology specimens, delays in processing, poor preservation, or inconsistent handling can damage RNA and reduce the accuracy of sequencing results.
Different types of samples need different preparation methods. There is no one-size-fits-all approach—procedures should be tailored based on the type of specimen, how it is preserved, and what type of RNA test is planned.
This review provides practical guidance. It outlines best practices for collecting, processing, and storing minimally invasive samples to help ensure reliable and reproducible RNA sequencing results in both clinical care and research.

## Introduction

Modern precision medicine increasingly uses RNA sequencing (RNA-seq) in clinical diagnostics and translational research, supporting high-throughput transcriptome analysis for applications such as gene expression profiling, detection of gene alterations and structural rearrangements, monitoring of treatment responses, and biomarker discovery. The increasing adoption of minimally invasive procedures, such as small biopsies, fine-needle aspiration (FNA), and exfoliative sampling, provides an effective way to obtain material for biomarker analysis, particularly for targets that are difficult to sample or that require repeat testing over time. Despite its growing use, especially in oncology, the success and reproducibility of RNA-seq depend highly on pre-analytical factors. These specimen types pose unique challenges, owing to their limited cellularity and increased susceptibility to degradation. Inconsistent handling, processing delays, and suboptimal preservation can substantially compromise RNA integrity, affecting downstream data quality and the biological interpretation of transcriptomic results. Given the variation in RNA quality requirements across different analytical approaches, a one-size-fits-all protocol is impractical. Instead, tailored standard operating procedures are needed, based on the specimen type, preservation method, and intended application.

To address a pressing need for fit-for-purpose guidance, several members of the Steering Committee for the NCI Notice of Funding Opportunity (NOFO) “Integrating Biospecimen Science Approaches into Clinical Assay Development” that included NCI scientists, current and former grantees, and extramural experts voluntarily formed a working group to identify pre-analytical best practices for RNA-seq from small biopsy, FNA, and exfoliation [1, 2]. In this review, the Working Group details each phase of the specimen lifecycle—from pre-procurement considerations (e.g., patient characteristics) to sample stabilization and storage—drawing from published literature and institutional practices. Emphasis is placed on the effects of preservation methods, such as formalin fixation and paraffin embedding (FFPE), as well as alternative stabilization strategies. While the focus of these recommendations is on messenger RNA, considerations relevant to the sequencing of other RNA species (e.g., microRNA, long non-coding RNA, ribosomal RNA) are also discussed. Our objective is to provide an evidence-based framework to support reproducibility, enhance standardization, and optimize resource utilization in research and clinical settings.

## Recording Biospecimen Data and Order Entry

Accurate and standardized documentation is critical for minimizing pre-analytical variability and ensuring the reliability of RNA-seq data. The essential metadata includes patient demographics such as age [3], sex [4], and diagnosis [5], as well as detailed information on specimen acquisition, including biopsy type [6], use of anesthesia [7], and labeling procedures. Documentation should also capture warm and cold ischemia time, stabilization method, storage conditions, and specifics of specimen processing and transport [8]. Consistent collection of this information improves quality control and comparability across studies. The Biospecimen Reporting for Improved Study Quality (BRISQ) checklist [8] and the Standard PREanalytical Code (SPREC) [9] for standardized biospecimen terminology and reporting provide comprehensive guidance on the types of data that should be collected.

## Biospecimen Types and Applications

While small biopsies and FNAs were traditionally obtained for diagnostic purposes and tumor subtyping, often relying heavily on immunohistochemistry, they are now frequently performed with the primary intent of obtaining material for molecular profiling [10]. Earlier integration of biomarker testing during the disease course has reduced the reliance on large surgical resections, which are often unavailable, particularly in patients with advanced or unresectable disease. As a result, minimally invasive sampling methods are now routinely used to support both diagnostic evaluation and predictive or prognostic biomarker analysis. This section provides an overview of the biospecimen types commonly used for RNA-based studies, with an emphasis on small biopsies, FNAs, and exfoliative cytology specimens (Table 1).

**Table 1:** Summary of studies on specimen preparation methods and RNA-seq outcomes

Article	Preparation method	Sample types	RNA quality assessment	RNA-seq outcome
Nibid, 2024 [6]	Surgical resection, biopsy, FNA cell block	FFPE; lung, bone, lymph node, adrenal, bladder	RT-qPCR, RNA-seq	RNA-seq success was highest in biopsy specimens. Surgical resections had moderate RNA-seq success, with wedge resections outperforming lobectomies. No successful RNA-seq from FNA cell blocks. RNA concentration differed by biopsy type, with thoracoscopy yielding the highest
Lin, 2024 [15]	FFPE scrolls vs sections	FFPE, frozen; lung, kidney, colorectal	Bioanalyzer	FFPE scrolls yielded higher RNA quality and more reliable RNA-seq outcomes than standard sections, suggesting scrolls are preferable for molecular testing
Sura, 2023 [38]	Cytospins fixed in Carnoy’s, 95% EtOH vs RNAlater^®^	Cytospins and frozen samples from breast effusions	Spectrophotometry, bioanalyzer	RNA quality remained stable for 7 days at 4 °C. RNA-seq (both targeted and whole transcriptome) showed high concordance with RNAlater^®^ controls. Carnoy’s fixed slides showed the best agreement with fresh-frozen in gene expression and mutation detection. All methods met input thresholds
Nagakubo, 2023 [159]	FFPE resection, FFPE cell block, cytology preparations	FFPE, cytologic samples from lung cancer	Bioanalyzer	Overall RNA-seq assay success rate was 98.2%. FFPE cell blocks and resections had higher success (>99%) than cytology samples (80%). RNA concentration did not predict success; DV200 was a better indicator. Microdissection was not necessary.
Marczyk, 2023 [35]	Carnoy’s, 95% EtOH, Diff-Quik cytosmears; frozen, FFPE	Cytosmears, frozen, FFPE from breast FNA and resections	Bioanalyzer	RNA-seq showed high expression concordance across preparation types. Despite lower DV200 in cytosmears and FFPE, targeted and whole transcriptome RNA-seq preserved clustering by patient
Sura, 2023 [39]	FFPE, 95% EtOH, Carnoy’s, Diff-Quik, ThinPrep, SurePath, RNAlater^®^	FFPE and cytology preparations vs frozen samples from breast effusions	Bioanalyzer	RNA quality was highest with PicoPure extraction. Diff-Quik and SurePath™ had lower DV200 compared with frozen. SETER/PR and PI3K signatures had highest concordance in Carnoy’s and Diff-Quik preps. FFPE blocks showed the lowest concordance and highest expression variability. Mutation detection remained robust across all types
Sura, 2025 [37]	Ficoll-Hypaque vs CytoLyt® for blood removal	Frozen and cytospin preparations of breast effusions	Bioanalyzer	Ficoll-Hypaque provided higher RNA integrity and longer fragment size. Both methods yielded successful RNA-seq, including accurate mutation and expression profiling using read-based and UMI-based library preparation methods. Ficoll preferred when higher fragment size is needed
Sura, 2025 [37]	RNAlater^®^ vs Carnoy’s fixed cytospins	Frozen vs cytospin slides from breast effusions	Bioanalyzer	Comparable RNA quality and RNA-seq performance. Both methods reliably detected gene expression markers. Cytospins proved valid for RNA-seq in clinical workflows, offering a viable alternative to frozen tissue
Newton, 2020 [144]	Microdissection vs macrodissection	FFPE; colorectal, breast, brain tumors	Transcript integrity number	RNA-seq expression profiles were highly correlated between replicate samples regardless of dissection method, indicating minimal impact of dissection strategy on transcriptome fidelity

*DV200* percentage of RNA fragments >200 nucleotides, *EtOH* ethanol, *FFPE* formalin-fixed paraffin-embedded, *FNA* fine needle aspiration, RNA-seq RNA sequencing, *RT-qPCR* reverse transcription-quantitative polymerase chain reaction, *UMI* unique molecular identifier

### Small Biopsies

Small biopsies refer to tissue samples collected using a wide needle, such as a core biopsy needle (typically 14–18 gauge), or other techniques such as a punch biopsy and a cryobiopsy. Such specimens, which offer a less invasive alternative to an excisional or surgical biopsy, are frequently used in oncology for diagnostic and molecular testing. Evidence suggests that the RNA yield and quality of core needle biopsies are comparable, and in some cases, even superior, to that obtained from larger resection specimens, depending on the procedure type, tumor type, and handling conditions [6, 11, 12]. Variability among biopsy specimens in next-generation sequencing (NGS) success and RNA yield and integrity is often influenced by the number of cores taken [13], the use of a coaxial needle system [13], the technical challenges of sampling tumors [13], and intrinsic tissue characteristics, such as cellularity, tumor stroma ratio, tumor viability and vascularity, and external factors such as cold ischemia time at room temperature [6, 14, 15].

For optimal RNA preservation and downstream sequencing applications, small biopsies should ideally be no thicker than 4–5 mm to ensure rapid and uniform penetration of fixatives or preservation agents [16–18]. This thickness facilitates proper molecular preservation while maintaining sufficient tissue architecture for histological evaluation. Additionally, for small biopsy specimens, individual fragments can be distributed across multiple blocks to minimize tissue depletion and optimize sample preservation. Post-FFPE processing can also affect RNA metrics. One study reported superior RNA quality in FFPE scrolls than that obtained from tissue sections [15]. Freshly cut FFPE sections should be processed immediately, ideally within 24 hours; however, evidence suggests that sections may retain acceptable RNA quality for up to 90 days at room temperature (e.g., 23–25 °C) [19–21].

### Cytopathology Specimens

Cytopathology specimens, including those obtained through FNA, exfoliative cytology, and even from supernatants, represent a valuable but historically underutilized resource for RNA-seq [22, 23]. Their minimally invasive nature and routine use in clinical diagnostics make them especially appealing for molecular applications when surgical specimens are unavailable or insufficient [23–25].

#### Fine-Needle Aspirations

Fine-needle aspirations involve the use of a thin needle (20–31 gauge) to sample cells from superficial or deep lesions. These procedures are commonly performed in outpatient settings with minimal risk and discomfort. While core biopsies preserve the architectural context of a lesion, which can be essential for certain histologic diagnoses [26], FNAs often yield higher tumor cellularity and purity by minimizing stromal contamination, making them especially valuable in oncologic molecular testing, where tumor cell enrichment can increase assay sensitivity [27, 28]. In the evaluation of bone lesions, FNA can complement or, in some cases, replace a core biopsy by providing diagnostic material that does not require decalcification, thereby preserving nucleic acid quality [29]. Fine-needle aspirations have been reported to produce sequencing metrics comparable to or surpassing those of a core needle biopsy [12, 23, 27, 30–33]. Fine needle aspiration specimens exhibit strong reliable performance in RNA-seq for transcriptomic profiling [34, 35].

#### Exfoliative and Liquid-Based Cytology Specimens

Exfoliative biospecimens comprise cells obtained via spontaneous shedding or mechanical collection (e.g., by brushing or scraping) from body fluids such as effusions, urine, cerebrospinal fluid, cyst fluid, or lavages. These samples are typically non-invasive, often available in large volumes, and can be preserved using various methods, including by freezing or as FFPE cell pellets, cytospin slide preparations, or using liquid-based cytology formats like ThinPrep^®^ (Hologic, Marlborough, MA, USA) and SurePath™ (Becton, Dickinson and Company, Franklin Lakes, NJ, USA). Liquid-based cytology and similar slide preparations generally preserve RNA integrity well, particularly when alcohol-based fixatives are used, and allow for the creation of multiple slides with uniform cell distributions [36–39].

Malignant effusion and other exfoliative cytology specimens are suitable for both targeted and whole transcriptome RNA-seq, with successful extraction and sequencing reported from cell pellets, direct smears, and liquid-based preparations [37–40]. Notably, a few studies have shown that, even in the absence of RNA-stabilizing preservatives, these specimens can retain sufficient RNA quality for transcriptomic analysis after prolonged refrigeration [38]. This resilience, particularly of samples stored as refrigerated cell pellets, underscores their potential utility in molecular profiling workflows, especially in settings where immediate processing is not feasible.

#### Supernatants

Supernatants—the fluid fraction remaining after centrifugation of FNA or exfoliative specimens (e.g., FNA rinses, effusions, cyst fluid, urine, cerebrospinal fluid, or biopsy collection fluid)—are often discarded in routine pathology workflows. However, these fluids can harbor clinically meaningful diagnostic material, including cell-free (cf) nucleic acids (cfDNA/cfRNA) and extracellular vesicles such as exosomes, and represent a valuable but underutilized resource for molecular testing, including RNA-seq [41–44].

Cytology sample-derived supernatants provide a complementary option for molecular profiling, particularly when the tissue or cellular component yields insufficient RNA for testing or when plasma-based liquid biopsies are limited by low cfRNA [45]. Cell-free RNA is found in several biological fluids, including blood, urine, bronchoalveolar lavage fluid, and effusion fluid [46, 47]. Compared to cfDNA, cfRNA is more labile, exhibits greater heterogeneity in fragment size, and is highly susceptible to degradation by ubiquitous RNases [48–50], but is more frequently protected through association with extracellular vesicles, protein complexes, or ribonucleoprotein particles [48, 50]. Analysis of cfRNA (or cfDNA) in cytology supernatants is also well suited for settings in which rapid turnaround time is desired [41]. These specimens have demonstrated clinical utility when identifying actionable alterations and offer a complementary approach that integrates features of both traditional tissue biopsies and plasma-based liquid biopsy testing [45]. However, because of its lability, cfRNA analysis necessitates more stringent pre-analytical controls and preservation strategies compared with cfDNA to ensure analytical reliability and reproducibility [47, 50].

## Collection of Tissue Specimens for RNA Analysis

### Ischemia Time

Pre-analytical collection intervals can affect RNA integrity. The warm ischemic time refers to the amount of time the tissue remains at body temperature with reduced or no blood supply before collection, while the cold ischemic time is the period between tissue removal and preservation, during which the specimen is kept cool or at room temperature but remains unstable. While prolonged periods of warm and cold ischemia can negatively impact RNA quality and downstream sequencing results, it is important to note that only surgical resection specimens and the samples derived from them experience warm ischemia, as small specimens collected via a core needle biopsy or FNA do not typically necessitate blood flow restriction during procurement. Thus, within the context of this review, only cold ischemia and its effects will be discussed. Tissue ischemia causes RNA degradation, leading to fragmentation and alterations in gene expression [15, 36, 38, 51–82], thus reducing data quality via the loss of low-abundance transcripts, introduction of transcriptomic bias, and skewing toward alternative splicing patterns [15, 38, 52–55, 57, 61]. Ischemia-induced stress responses activate genes related to inflammation, apoptosis, and hypoxia, further distorting the transcriptome from its original in vivo state and complicating downstream analysis and data interpretation [72, 80, 83].

Currently, expert opinion and consensus guidelines recommend immediate processing or preservation of tissue specimens, with an optimal ischemia time of <20 minutes to 1 hour [17, 84]. It has been reported that RNA-seq quality is preserved in tissues stored at 4 °C for less than 24–48 hours or at 25 °C for less than 0.5–2 hours, while longer cold ischemia times reduced RNA integrity and sequencing outcomes [15, 38, 52, 54, 55, 57, 61]. Storing specimens on wet ice can delay cold ischemia-induced RNA degradation [15, 53, 61]. Although studies differ on when pre-fixation delays begin to affect RNA quality, with a range from minutes to several hours, the impact is highly variable and depends on factors such as specimen type, cellular composition, preservation method, and the tissue collection technique [15, 38, 52–55, 57, 61]. Nonetheless, RNA quality metrics, including RNA integrity number (RIN), generally decline as cold ischemia time lengthens, indicating RNA degradation and potentially altering the reliability of downstream analysis, including that of RNA-Seq [15, 38, 52–55, 57, 61]. RNA-sequencing specific studies on cold ischemia time are highlighted in Table 2.

**Table 2:** Summary of studies on specimen ischemia times and RNA-seq outcomes

Article	Ischemia conditions	Sample types	RNA quality assessment	RNA-seq findings
***Cold ischemia: time and temperature***				
Lin, 2024 [15]	4 °C: <0.5–48 h 25 °C: <0.5–48 h	Surgical resections (lung, kidney, colorectal); FFPE, frozen	Bioanalyzer	RNA-seq performance was optimal when tissues were kept at 4 °C for <48 h or at 25 °C for <0.5 h. Longer cold ischemia times led to reduced RNA integrity and sequencing performance
Sura, 2023 [38]	4 °C: 0, 1, 4, 7 days	Breast effusion cytospins (Carnoy’s or EtOH fixed); frozen	Bioanalyzer	Gene expression and mutation detection via targeted and whole transcriptome RNA-seq remained highly reproducible up to 7 days of storage at 4 °C. Carnoy’s fixed slides demonstrated superior stability in transcript detection. All preparations met quality and input thresholds for RNA-seq
van der Wijngaart, 2023 [52]	RT: 5 min, 2 h	CNB, cell lines (liver, neoplastic, normal); frozen	Bioanalyzer	RNA-seq outcomes were not impacted by up to 2 h of cold ischemia at room temperature. Expression profiles clustered more by biological source (patient ID) than by ischemia duration or preservation method, indicating robust expression stability under these conditions
Madissoon, 2019 [54]	4 °C: 0–72 h in HypoThermosol^®^	Surgical and cell line samples (lung, spleen, esophagus); frozen	Bioanalyzer	Cold ischemia ≤24 h maintained RNA integrity, cell viability, and sequencing quality for both bulk RNA-seq and single-cell RNA-seq. Delays up to 72 h in HypoThermosol^®^ did not significantly alter gene expression profiles or clustering, supporting extended cold preservation
Jones, 2019 [55]	RT: 1–12 h in a humidified chamber	Surgical resections (colon, kidney, ovary); FFPE, frozen	Bioanalyzer	Delay-to-fixation times up to 12 h had minimal effect on RNA-seq results. However, increased variability in miRNA expression and elevated abundance of small RNA fragments were observed with longer ischemia, indicating selective sensitivity of small RNA populations
Sun, 2016 [61]	Ex vivo ischemia: <15 min to 4 h; kept at room temperature, on ice, or frozen	Kidney resections (neoplastic and normal); frozen, fresh	Bioanalyzer	RNA remained stable for up to 4 h on ice but degraded after 1 h at room temperature or 4 h in saline. Despite high RINs, RNA-seq showed altered expression of hypoxia/stress-response genes post-surgery. This highlights the importance of tightly controlled ischemia intervals to preserve transcriptomic fidelity
***Warm ischemia time***				
Zhang, 2019 [57]	≤3 h vs >3 h (cold ischemia ≤20 min vs 20–40 min)	Gastric surgical biopsies; frozen	Bioanalyzer	RIN values did not correlate with ischemia time. However, RNA quality correlated positively with tumor cell percentage and negatively with stromal content. RNA-seq profiles remained stable, but sample cellularity had more impact than ischemia conditions

*CNB* core needle biopsy, *EtOH* ethanol, *FFPE* formalin-fixed paraffin-embedded, *h* hours, *min* minutes, *miRNA* microRNA, *RIN* RNA integrity number, *RNA-seq* RNA sequencing, *RT* room temperature

The composition of exfoliative biospecimens is highly variable, and no universally accepted guidelines exist for their ischemia time or delay to fixation. Many effusion and other fluid samples are nutrient rich, allowing cells to remain viable for extended periods post-collection [85]. However, it is generally recommended that laboratories process effusion samples promptly or store them at 4 °C until processing [40, 85]. While the data on the effects of delayed fixation of exfoliative biospecimens on RNA integrity are limited, studies suggest that cellular morphology, immunoreactivity, and nucleic acid integrity, including that of RNA, can remain stable for days to weeks without preservatives when refrigerated [38, 86–88].

## Sample Processing and Preservation

In cases where immediate analysis of a biospecimen is not possible, it is essential to choose the appropriate method of preservation. Common tissue preparation methods include the RNAlater^®^ technique [89, 90], formalin fixation [15, 16, 18, 19, 34, 35, 51, 53, 55, 72, 74, 83, 90–105], freezing of nitrocellulose imprints [106–110], and snap-freezing [111] with or without optimal cutting temperature compound (OCT) [53, 82, 97, 112, 113] (Table 3). While preserving specimens by RNAlater^®^ [89, 90] or snap-freezing [114] may be preferable for RNA-seq analysis, owing to superior RNA stabilization, alternative preservation methods, such as FFPE, OCT, and nitrocellulose imprints [115], may be more suitable when evaluation of other analytes is anticipated. While calcified tissue may require additional processing prior to sequencing, decalcification has been negatively correlated with RNA yield and significantly lower NGS success rates (54 vs 92%) [116]. Strong acids such as hydrochloric acid and nitric acid should be avoided as they increase RNA degradation [17, 28, 117]. When necessary, chelating agents, such as EDTA-based decalcification, are preferred for small bone biopsies when nucleic acid analysis is anticipated [17, 28, 118].

**Table 3:** Summary of studies on specimen preservation type and RNA-seq outcomes

Article	Preservation variable	Sample types	RNA quality assessment	Detailed RNA-seq findings
***Formalin concentration***				
Hatanaka, 2024 [125]	10% vs 15% (fixation time <48 h vs 48–72 h)	Thyroid FFPE	Bioanalyzer, PCR	Neither concentration nor fixation time consistently affected RNA integrity or RNA-seq success. However, ΔCq values were strongly predictive of sequencing quality
***Time in formalin***				
Nibid, 2024 [6]	1–6 days	Lung, bone, lymph node, adrenal, bladder; FFPE	PCR, Myrapod RNA panel	RNA degradation increased with longer formalin fixation, leading to decreased RNA-seq success. Higher fragmentation index and RNA concentration predicted better outcomes. Samples fixed beyond 3 days showed reduced performance
Lin, 2024 [15]	12 h, 24 h, 48 h at 25 °C	Same as above	Bioanalyzer	48 h of formalin fixation at room temperature yielded the best RNA integrity and RNA-seq performance among tested conditions
Amemiya, 2023 [126]	1–240 h	Liver FFPE, microdissected	Bioanalyzer, fluorometry	RNA yield, DV200, and gene expression reproducibility dropped significantly after 48 h. Longer fixation reduced library complexity and internal control gene detection
***FFPE vs frozen or fresh***				
Lin, 2024 [15]	FFPE vs frozen vs paraformaldehyde	Lung, kidney, colorectal	Spectrophotometry, bioanalyzer	Expression biases were observed in FFPE compared with frozen, including altered housekeeping gene levels. Despite these differences, FFPE RNA-seq data remained suitable for expression analysis, though gene-level discrepancies were present
Jacobsen, 2023 [94]	FFPE vs frozen vs fresh (fixation time: 23–97 h)	Heart FFPE	Bioanalyzer, PCR	FFPE preservation led to increased intergenic content and variability. Fixation time did not affect gene expression outcomes, but preservation type was a major determinant of sequencing quality
Skaftason, 2022 [95]	FFPE vs frozen	DLBCL lymph nodes	Bioanalyzer	Despite significantly degraded RNA and lower mapping rates in FFPE, gene expression profiles remained strongly correlated with frozen samples. FFPE data were sufficient for transcriptomic analyses
Boneva, 2020 [96]	FFPE vs fresh	Eye (conjunctiva)	Bioanalyzer	FFPE samples supported comprehensive transcriptome profiling via MACE RNA-seq. Despite higher multi-mapped reads and lower diversity, gene detection and distribution mirrored fresh tissue
Newton, 2020 [144]	FFPE vs frozen	CRC, breast, brain	Transcript integrity number	Whole transcriptome correlation was high (r = 0.95). Some transcript classes (e.g., zinc finger genes) were underrepresented in FFPE, but exome-level agreement reached 98%
Jones, 2019 [55]	FFPE vs frozen	Colon, kidney, ovary	Bioanalyzer	Expression differences were mainly tissue-specific. FFPE samples had more intronic reads, impacting splice variant interpretation. UTR signal correlation with frozen was high
Li, 2014 [99]	FFPE vs frozen	Kidney	Bioanalyzer, PCR	Expression of RCC-related transcripts showed strong correlation. Profiles clustered by patient, confirming comparability
Hedegaard, 2014 [100]	FFPE vs frozen	Bladder, prostate, colorectal	Bioanalyzer	FFPE had lower RNA quality and yield. Differentially expressed genes overlapped but were more numerous in FFPE, likely because of fixation artifacts
Norton, 2013 [101]	FFPE vs frozen	Breast cancer cell lines	Bioanalyzer	RNA-seq from FFPE samples enabled fusion, SNV, and expression analysis with strong reproducibility despite lower sensitivity
Meng, 2013 [102]	FFPE vs frozen	Breast, kidney, muscle	PCR	miRNA profiles were concordant overall. FFPE samples had fewer canonical-sized RNAs, but major miRNAs were reliably detected
Weng, 2010 [105]	FFPE vs frozen	Kidney	Bioanalyzer, PCR	miRNA expression showed good agreement between FFPE and frozen samples. Differentially expressed miRNAs overlapped across methods
***FFPE vs RNAlater***				
Sorokin, 2024 [93]	FFPE vs RNAlater^®^	Colon resections	Tapestation	Fusion detection performance was comparable between FFPE and RNAlater^®^-preserved tissues, with similar total fusion counts. FFPE samples had longer insert sizes and higher uniquely mapped read fractions. Patient-to-patient variability influenced fusion detection more than the preservation method
Marczyk, 2019 [34]	FFPE vs RNAlater^®^	Breast FNA/resection	Bioanalyzer	FFPE had lower RNA integrity but higher TIN. Gene expression in highly expressed genes was strongly concordant; low-expressers showed more bias in FFPE
Li, 2018 [98]	FFPE vs RNAlater^®^	Breast	Bioanalyzer	FFPE showed lower RINs and more intronic reads, but hormone receptor-based clustering remained intact. Expression profiling was still feasible
Eikrem, 2016 [90]	FFPE vs RNAlater^®^	Kidney	Bioanalyzer	Despite poorer integrity, FFPE supported accurate gene expression Clustering reflected disease status (tumor vs normal), not preservation type
***FFPE vs PAXgene***				
Groelz, 2013 [103]	FFPE vs PAXgene	Rat organs	Bioanalyzer	PAXgene-fixed tissues preserved RNA quality better than FFPE. RNA-seq and RT-PCR performance closely resembled fresh frozen
***Snap-freezing techniques***				
van der Wijngaart, 2023 [52]	LN_2_ vs snap freezer	Liver biopsies	Bioanalyzer	No significant differences in RNA-seq profiles were found between freezing methods. Expression patterns clustered by patient identity rather than preservation strategy
Fang, 2023 [114]	Snap freezing ± isopentane	Skull resections	Bioanalyzer	Isopentane-free snap freezing significantly improved RNA integrity and tissue morphology, enabling higher quality RNA-seq data
***Cytospin preservation methods***				
Sura, 2023 [38]	Carnoy’s vs ethanol vs RNAlater^®^	Breast effusion cytospins	Bioanalyzer	Carnoy’s-fixed slides showed better expression concordance with RNAlater^®^ than ethanol-fixed ones. Ethanol fixation resulted in reduced PI3K signature expression and increased expression variability, particularly for the SETER/PR signature
Marczyk, 2023 [35]	FFPE, Carnoy’s, ethanol, Diff-Quik, RNAlater^®^	Breast FNA, resections	Bioanalyzer	Diff-Quik-stained smears were unsuitable for RNA-seq. Despite lower RNA quality in FFPE and cytosmears, gene expression profiles and variant allele frequencies remained consistent across all preservation methods

*ddCq* comparative delta Cq, *DV200* percentage of RNA fragments >200 nucleotides, *FFPE* formalin-fixed paraffin-embedded, *FNA* fine needle aspiration, *h* hours, *LCM* laser capture microdissection, *miRNA* microRNA, *PCR* polymerase chain reaction, *RIN* RNA integrity number, *RNA-seq* RNA sequencing, *RT-PCR* reverse transcription-polymerase chain reaction, *SNV* single nucleotide variant, *TIN* transcript integrity number, *UTR* untranslated region, *ΔCq* change in quantification cycle

### Snap-Freezing

Snap-freezing (flash-freezing) in liquid nitrogen (LN₂) or the vapor phase of liquid nitrogen (VPLN) is widely recognized as the gold standard for preserving RNA in tissue specimens. To maintain optimal RNA integrity for RNA-seq analysis, fresh tissues should be promptly processed, ideally by dissection into small fragments and freezing within 0.5–2 h after excision [17, 84]. While the freezing rate of the specimen is determined by several parameters (including cold source, coolant, cryogenic medium, and material of the storage container) [119], an ideal snap-freezing method for RNA-seq samples ensures rapid and uniform freezing of tissues between − 53 and – 93 °C, thus minimizing RNase protein activity [120]. While snap-freezing tissue directly in LN_2_ is the coldest option (− 196 °C), its rapid cooling rate can cause ice-crystal formation, and the low boiling point of LN_2_ can create an insulating vapor barrier when it encounters a tissue specimen at ambient temperature, leading to uneven freezing of the specimen’s periphery and core, thereby affecting its morphological integrity. Snap-freezing tissue in VPLN minimizes intracellular ice crystal formation, thereby limiting the mechanical disruption of the tissue, while also suppressing RNase-mediated RNA degradation [111]. For effective snap-freezing in VPLN, specimens ≤ 0.4 cm in diameter should be placed in cryogenic specimen storage containers, tightly sealed, and rapidly frozen (i.e., in under 2 min) [111]. Alternatives include direct immersion in LN₂ or freezing on dry ice if LN₂ is not available; however, these methods are discouraged for specimens intended for morphological analysis unless used in combination with a cryoprotectant such as OCT. Isopentane is commonly used to prevent the formation of artifacts; however, specimens snap-frozen by immersion in pre-cooled isopentane exhibited lower RNA quality and RNA-seq performance than those frozen using other methods [114]. Comparable RINs and indistinguishable RNA-seq gene expression profiles were obtained between specimens (of cancer cell lines and normal liver biopsies) snap-frozen at −196 °C in LN_2_ or at – 73 °C using an electronic device with an adjustable cold sink temperature [52]. Further, paired tumor specimens snap-frozen in isopentane pre-cooled with dry ice, or at – 80 °C with a coolant-chilled freezing device, exhibited comparable RNA yield, integrity, and RNA-seq quality control parameters [121]. Therefore, RNA-seq analysis may be insensitive to minute differences introduced by different snap-freezing rates and temperatures, as long as the freezing is at or below – 70 °C.

Embedding tissue in OCT compound prior to freezing is common in clinical practice and can be used for preserving both tissue morphology and RNA integrity [53, 82, 97, 112, 113]. Optimal cutting temperature compound, a water-soluble glycol and resin matrix, provides structural support during cryosectioning and acts as a cryoprotectant by minimizing ice crystal formation during freezing. To ensure even cooling and reduce the risk of cracking, tissue embedded in OCT is typically snap-frozen using VPLN, precooled isopentane (2-methylbutane), or other rapid freezing methods. Notably, methanol, ethanol, or acetone are unsuitable substitutes for isopentane, as an interaction between OCT and these solvents prevents proper freezing at −20 °C. Embedding in OCT is particularly useful for fragile tissues susceptible to mechanical damage, maintaining RNA integrity comparable to standard snap-freezing. Removal of excess OCT prior to RNA extraction is critical, as residual compound may interfere with downstream molecular techniques such as reverse transcription-polymerase chain reaction [113], microarray [122], and RNA-seq [123].

When properly handled, fresh-frozen tissues can consistently produce high-quality RNA. Snap-frozen tissues yield significantly higher RNA quality and support more robust and reliable downstream gene expression analysis than FFPE-prepared specimens (Table 3) [15, 34, 51, 53, 55, 93–95, 97, 98]. Overall, the literature suggests that acceptable and comparable RNA-seq results can be obtained regardless of whether snap-freezing is achieved using LN₂ immersion or a snap-freezing device [52, 114].

### RNAlater^®^ and Proprietary RNA Preservative Media

RNAlater^®^ (Thermo Fisher Scientific, Waltham, MA, USA) is a more commonly used proprietary tissue preservation solution formulated to stabilize and protect RNA by inhibiting endogenous RNase activity. It allows for flexible sample handling, enabling tissues to be stored at ambient temperature for short durations (up to 72 hours) or at refrigerated and frozen temperatures for longer term preservation, without significant RNA degradation. RNA isolated from tissues preserved in RNAlater^®^ and kept at room temperature or frozen consistently exhibits similar RNA quality and gene expression profiles to those extracted from FFPE-treated [89, 90] or snap-frozen [124] specimens. It is worth noting that a variety of commercial reagents (e.g., KeepRNA™, RNAstorm™, TRIzol^®^) are available. These stabilizing agents help maintain nucleic acid integrity during collection, storage, and transport; however, they differ in their compatibility with specific specimen types, storage conditions, and downstream molecular workflows, and therefore must be selected in a fit-for-purpose manner. For specimens collected for commercial molecular testing (e.g., Afirma^®^ or PancreaSeq^®^), the appropriate manufacturer-recommended collection and transport media should be used, ideally at the time of specimen collection.

### Formalin Fixation

The FFPE tissue block remains the most widely used method for tissue preservation in pathology. Although formalin fixation can degrade RNA, FFPE samples are often the only material available, especially in clinical practice, and can still be suitable for RNA-seq using optimized extraction and analysis protocols. Formalin fixation induces extensive crosslinking of DNA and RNA, leading to nucleic acid fragmentation and chemical modifications such as base methylation and sequence alterations. These changes can introduce significant artifacts, complicating downstream molecular applications such as polymerase chain reaction [17], NGS [15, 17], and gene expression analysis [15, 16, 18, 19, 34, 35, 51, 53, 55, 72, 74, 83, 90–105].

Formalin concentration is a predictor of NGS success, and most guidelines and experts recommend using 10–15% neutral phosphate-buffered formalin at pH 7.0 [17, 19, 125]. Limiting formalin fixation to a window of 6–24 hours at room temperature allows for optimal tissue preservation with minimal molecular degradation; fixation beyond 24 hours is associated with reduced RNA yield, increased RNA fragmentation [15, 17, 51, 74], altered transcript distribution impacting splicing distribution [15], increased off-target (non-exonic) reads [15, 94], poorer preservation of 5′ and 3′ ends than of mid regions [74], discordant DNA-RNA variant calls [94], and potentially fewer mappable sequencing reads [126]. To ensure uniform and adequate tissue penetration, a minimum formalin-to-tissue mass ratio of at least 4:1 is advised [17, 125]. Embedding the fixed tissue in high-quality, low-melting-point paraffin further preserves sample integrity and facilitates reproducible sectioning [16, 17, 19, 74].

### Cytopathology Preparation

Direct smears prepared from FNAs are typically fixed using alcohol-based solutions (e.g., 95% ethanol or modified Carnoy’s solution) and stained via Papanicolaou staining or air-dried and stained via a Romanowsky method. Both alcohol-fixed and air-dried cytology slide preparations can yield RNA of sufficient quality and quantity for targeted and whole transcriptome RNA-seq [35–39, 127]. Unlike FFPE cell blocks, cytological slide preparation does not involve formaldehyde-induced crosslinking, RNA fragmentation, and chemical modification, enabling better preservation of nucleic acids for sequencing applications. Cytology preparation (e.g., direct smears or cytospins) may in fact lead to better RNA integrity and sequencing success than FFPE cell blocks, especially when specimen size is limited or tumor cellularity is low [23, 25, 39, 41, 116, 128, 129]. Importantly, in scenarios where FFPE cell block or tissue specimens are insufficient or exhausted, archived stained cytology slide preparations can provide a reliable alternative for RNA extraction [116]. Before RNA extraction from stained smears, the coverslip must be removed [39]. This can be achieved by freezing methods [130, 131] or soaking slides in xylene [39]; however, the use of heat or open flame should be avoided [28].

Cell blocks are created by concentrating cells from FNA or exfoliative samples into pellets via centrifugation, followed by FFPE, allowing compatibility with existing FFPE workflows and validation protocols. While cell blocks are widely used for DNA-based testing, RNA extracted from FFPE cell blocks tends to be more degraded (via crosslinking and fragmentation) than that from non-formalin-fixed cytology specimens. This degradation can impact RNA-seq applications, particularly those requiring full-length transcripts or sensitive detection of fusions [35, 39]. Despite these limitations, using optimized extraction protocols and short fixation durations can mitigate RNA degradation.

### Nitrocellulose Tissue Printing

In nitrocellulose tissue printing, fresh tissue specimens such as core biopsies are gently pressed onto a nitrocellulose membrane, facilitating the transfer of cellular biomolecules such as RNA, DNA, and proteins onto the membrane surface [106–110]. Once imprinted, the membranes are snap-frozen, typically on dry ice or in LN_2_, and stored at −80 °C until processing. This minimally invasive approach to harvesting high-quality nucleic acids avoids consuming or damaging the original tissue core, which is thus preserved for essential histopathological diagnosis [115, 132]. Nitrocellulose tissue prints allow for rapid stabilization of nucleic acids, enabling immediate downstream molecular applications. The material collected on the membrane can be processed for RNA and DNA extraction, with yields and integrity suitable for gene expression profiling, genotyping, and advanced sequencing-based assays such as RNA-seq [115]. This technique is particularly advantageous in clinical and research settings where tissue availability is limited, as it maximizes molecular data acquisition while conserving tissue architecture for diagnostic evaluation.

## Tissue and Cellular Sample Storage

Storage of FFPE blocks can lead to cumulative RNA degradation, with reductions in quality and yield [6, 14, 35, 94, 96, 98, 100, 102, 125, 126], particularly of messenger RNA, observed even after 30 days of storage [6] (Table 4). The extent of degradation is influenced by both intrinsic factors, such as sample type [6], and by processing-related parameters related to the effectiveness of dehydration and formalin fixation [15]. RNA quality generally declines with longer storage, especially beyond 2–3 years [14, 15, 35, 95, 96, 100, 125, 126]. RNA from older FFPE blocks may still be suitable for targeted NGS panels, depending on amplicon length and RNA quality, but severely degraded samples can often result in failure during library preparation or amplification [6, 15].

**Table 4:** Summary of studies on specimen storage duration and temperature and RNA-seq outcomes

Article	Storage condition	Sample types	RNA quality assessment	Detailed RNA-seq findings
***Room temperature storage***				
Nibid, 2024 [6]	<30 d, 31–60 d, >60 d	Lung, bone, lymph node, adrenal, bladder; FFPE	PCR	RNA fragmentation index declined with increasing storage time. Samples stored <30 da showed better RNA-seq success (68%) compared with those stored >60 days (38%). Longer storage correlated with lower RNA quality and RNA-seq failure
Watanabe, 2024 [14]	<1 y to ~5 y	Lung, colon, uterus, soft tissue; FFPE	Bioanalyzer	RNA quality declined gradually over time. ddCq and DV200 decreased, especially beyond 3 y. Q-value did not consistently reflect RNA degradation
Hatanaka, 2024 [125]	<1 y to <10 y	Thyroid; FFPE	Bioanalyzer, PCR	RNA-seq and PCR success declined after 2–3 y. RNA DV200 and ΔCq values fell after 2 y; RNA success dropped to 78% in blocks aged 9–10 y
Amemiya, 2023 [126]	500 d	Liver; FFPE (LCM)	Bioanalyzer, spectrophotometry, fluorometry	Extended storage at room temperature resulted in reduced RNA yield, DV200, and RNA >200 nucleotide content, impairing RNA-seq data quality
Skaftason, 2022 [95]	1–11 y	Lymph node; FFPE, frozen	Bioanalyzer	No observable relationship between FFPE block age and RNA DV200, suggesting long-term storage did not impact RNA-seq quality
Boneva, 2020 [96]	56 d to 13.4 y	Eye (conjunctiva); FFPE, fresh	Bioanalyzer	Longer storage reduced uniquely mapped reads (from 70 to 35%), but overall gene detection (~18,000) and library diversity remained unaffected. No clear link between storage time and gene expression count
Li, 2014 [99]	2 y	Kidney; FFPE, frozen	Bioanalyzer, PCR	RNA-seq results from FFPE samples stored 2 y correlated well with fresh frozen samples. Transcriptome clustering and differential expression remained reliable
Hedegaard, 2014 [100]	2 mo to 20 y	Multiple tissues; FFPE, frozen	Bioanalyzer	RNA quality declined over time, affecting library construction and mapping. Variant overlap with frozen remained high up to 3 y but degraded with age. Low-frequency mutations were still reliably detected
Hedegaard, 2014 [100]	2–13 y	Bladder, prostate, colorectal; FFPE, frozen	Bioanalyzer	Gene expression was strongly correlated between FFPE and frozen samples. No consistent RNA-seq degradation linked to storage time
Meng, 2013 [102]	2–9 y	Various; FFPE, frozen	PCR	miRNA detection rates were stable across storage durations. No significant association between miRNA expression levels and sample age
Weng, 2010 [105]	1–8 y	Kidney; FFPE, frozen	PCR	miRNA profiles remained consistent over long-term storage. Storage duration had no measurable impact on expression reliability
***80 °C Storage***				
Marczyk, 2023 [35]	15–528 d	Breast FNA/resection; FFPE, frozen	Bioanalyzer	Storage time weakly correlated with RIN/DV200 in cytosmears, but not in FFPE or frozen samples. RNA-seq outcomes remained stable across storage durations
***Storage temperatures compared***				
Lin, 2024 [15]	4 °C vs 25 °C	Lung, kidney, colorectal; FFPE, frozen	Bioanalyzer	RNA degradation was slower at 4 °C. FFPE blocks stored at 25 °C degraded faster, impacting RNA-seq performance. Cold storage recommended
Jacobsen, 2023 [94]	20–42 d (variable temperature)	Heart; FFPE, frozen	Bioanalyzer	Short-term storage had minimal impact on RNA-seq. Preservation type influenced sequencing outcomes more than storage time. Expression profiles were consistent across methods

*d* days, *ddCq* comparative delta Cq, *DV200* percentage of RNA fragments >200 nucleotides, *FFPE* formalin-fixed paraffin-embedded, *FNA* fine needle aspiration, *LCM* laser capture microdissection, *miRNA* microRNA, *mo* months, *PCR* polymerase chain reaction, *Q-value* false discovery rate-adjusted *p* value, *RIN* RNA integrity number, *RNA-seq* RNA sequencing, *y* years, *ΔCq* change in quantification cycle

RNA integrity is highly influenced by the sample storage temperature. Room temperature storage (24 °C) of FFPE blocks can lead to significant RNA degradation, reduced RINs, and diminished transcript detection [15, 126]. Storage at −80 °C consistently preserves RNA yield and quality for several years [53, 133]. RNA from tissue specimens stored at −140 °C remained stable for up to 7 years, showing no substantial decline in quality [112].

Storage conditions have been less well studied for effusion samples than for tissue specimens. The RNA in non-preserved effusion fluids refrigerated at 4 °C for up to 7 days may be stable and suitable for RNA-seq [38]. Exosomal microRNA from malignant effusions and supernatants has been successfully sequenced after storage at −70 °C [134]. Cell-free microRNA appears to remain stable in effusion fluid at room temperature for up to 24 hours and is resilient to multiple freeze-thaw cycles prior to extraction [135].

## Determining Specimen Adequacy

Confirming adequate cellularity is essential for both morphologic diagnosis and biomarker testing. While the tumor cellularity of a specimen directly affects the sensitivity of variant detection, copy number analysis, and other molecular signatures, defining a minimum tumor cellularity threshold using a one-size-fits-all approach is precluded by assay-specific cellularity requirements. Complicating matters further, visual estimates are often inconsistent between observers, tending to overestimate tumor relative to benign components, with some early reports also noting poor concordance between pathologist estimates and computational or artificial intelligence (AI)-based assessments [124, 136, 137]. Recent work has demonstrated that digital pathology tools with AI support—including open-source approaches—can provide consistent and reproducible tumor cellularity measurements across surgical specimens, small biopsies, and cytology cell blocks [137–139]. Compared with unaided human review, these computational methods tend to yield lower, more accurate tumor estimates and reduce observer bias [137–139]. Incorporating digital and AI-based tools into routine molecular pathology may therefore improve the reliability and reproducibility of sequencing results from limited biospecimens by providing a consistent assessment of tumor cellularity.

### Rapid On-Site Evaluation

Rapid on-site evaluation, commonly used in cytopathology for FNA biopsies, allows for immediate intraprocedural sample assessment [140]. A glass-slide aspirate cytosmear sample can be microscopically reviewed immediately following collection to evaluate material adequacy and tumor cellularity [140]. Rapid on-site assessment provides real-time feedback to the proceduralist, ensuring that the sample is sufficient for downstream molecular testing and helping prevent the need for repeat biopsies [140]. The cell block, stained aspirate cytosmear, or supernatant fluid can also be used for later RNA extraction and sequencing [39].

### Touch Preparation

This method can be used to evaluate the cellular composition of a core biopsy or tissue sample, typically as an immediate intra-procedural assessment during the procedure [140, 141]. The technique involves lightly pressing the freshly procured specimen onto a glass slide to transfer cells onto the surface, which are then examined microscopically. The value of touch preparations for core biopsy specimens depends largely on the type of tissue being analyzed [141]. While this method is highly effective for rapidly assessing certain tissue types, including most solid tumors and hematolymphoid neoplasms, it exhibits limited utility for other types, such as fibrous or cystic lesions [141]. While it can be useful for preliminary assessment during rapid onsite evaluation, the depletion of core tumor cells can be a drawback of this method.

### Sample Microdissection and Macrodissection

Both microdissection and macrodissection are techniques used to isolate specific cellular components from a tissue sample for analysis. Microdissection, which can be performed manually using a microscope and enhanced by using tools such as laser capture or image processing software, improves precision in tissue targeting. It minimizes contamination from the surrounding non-target tissue and enriches the desired cell type in the sample. In contrast, manual microdissection, while less precise, is more time efficient, cost effective, and supports high throughput [142]. Both methods of tumor enrichment are recommended to ensure that sample inputs meet the minimum tumor cellularity and RNA input required for RNA-seq assays [143, 144].

## RNA Extraction and Storage

Selecting the appropriate RNA extraction method or kit requires consideration of the biospecimen type, intended downstream application, and the quality, quantity, and type of RNA required. The various methods, which are optimized based on sample characteristics (e.g., fresh or fixed tissue, low-input material, or biofluids), vary in their ability to recover intact or fragmented RNA. The quality of RNA required depends heavily on the intended application. For instance, whole transcriptome RNA-seq requires large amounts of high-quality RNA, while more fragmented RNA may be acceptable for targeted RNA-seq [145, 146].

Although data specifically addressing the effects of storage duration and conditions on RNA-seq results from RNA isolated from small biopsy and cytology specimens remain limited, recommendations to minimize RNA degradation can be extrapolated from broader investigations. An excellent comprehensive review was recently published that recommends the following based on a detailed accounting of interactions between external factors and RNA stability; RNA should be eluted in RNase-free water, Tris-EDTA buffer prepared with DEPC-treated water, or an appropriate stabilization solution, and aliquoted into single-use volumes to minimize freeze-thaw-associated degradation; aliquots should be stored in air- and water-tight tubes at or below – 80 °C in complete darkness. [147]. Notably, intrinsic factors affecting primary, secondary, and tertiary structures of the RNA molecule [147]; the tissue source [148]; and the concentration of the RNA sample [148] may impart effects and introduce variability in RNA stability. Short-term storage (days to weeks) at – 20 °C is generally acceptable, whereas long-term storage should be performed at – 70 °C to – 80 °C [149]. Repeated freeze–thaw cycles, prolonged bench exposure, and inappropriate storage buffers accelerate RNA degradation and can reduce library complexity, transcript detection, and reproducibility in RNA-based assays [149].

## RNA Quality Metrics

RNA quality assessment typically encompasses RNA quantity, purity, and integrity. RNA quantity is measured via spectrophotometry, using absorbance at 260 nm (A260) to estimate concentration. Purity is assessed using the A260/A280 and A260/A230 ratios, which indicate protein and salt/phenol contamination, respectively; ideal values for both are close to 2.0.

RNA integrity has traditionally been evaluated using the 28S/18S ribosomal RNA ratio, although this approach is now largely considered unreliable [150]. This has been replaced by the RNA integrity number, generated via capillary electrophoresis platforms such as the Agilent Bioanalyzer, TapeStation, or Fragment Analyzer (Agilent Technologies, Santa Clara, CA, USA), which has become the standard metric. RNA integrity number scores range from 1 (highly degraded) to 10 (intact), with values ≥7 generally accepted as reflecting high-quality RNA for downstream applications such as RNA-seq. However, the RNA integrity number primarily reflects ribosomal RNA integrity and does not necessarily correlate with messenger RNA quality or capture the degradation caused by pre-analytical factors such as ischemia, fixation, or prolonged storage [151–155].

To address these limitations, alternative or complementary metrics have been adopted. The DV200 metric, the percentage of RNA fragments longer than 200 nucleotides, has proven especially useful for FFPE and degraded samples [154, 155]. DV200 values >70% are typically considered acceptable for RNA-seq library preparation. Another post-sequencing metric, the transcript integrity number—introduced in 2016 within the RSeQC toolkit [156]—quantifies the uniformity of read coverage across the length of gene bodies, based on the premise that degraded RNA yields 3′-biased coverage owing to its fragmentation. Unlike RIN, a pre-sequencing measure of ribosomal RNA quality, transcript integrity number allows retrospective evaluation of messenger RNA integrity in sequencing data. Importantly, certain library preparation methods, such as exome-capture RNA-seq, can generate evenly distributed reads from degraded RNA, resulting in high transcript integrity number scores even when RIN is low.

## Clinical Framework Behind RNA Management of Routine Diagnostic Samples

In clinical practice, choosing an appropriate molecular testing strategy depends on several interrelated factors, including the clinical indication for testing, disease type, specimen adequacy and availability, and the intended use of the data (e.g., diagnosis, prognosis, therapeutic selection, or disease monitoring). The first step is to define the clinical goal of testing. RNA-based assays may be ordered to support initial diagnosis (e.g., fusion-driven tumors), guide treatment decisions (e.g., targetable or expression-based biomarkers), provide prognostic or predictive information, or monitor disease evolution and resistance mechanisms. Close communication between the molecular pathology laboratory and ordering providers—including oncologists, surgeons, interventional radiologists, and genetic counselors—together with recommendations developed by professional societies, is needed to ensure that the selected assay is clinically appropriate and that specimen handling is optimized for the intended use [157]. Given the inherent differences in analyte stability and input requirements between DNA- and RNA-based testing, a practical clinical strategy is to adopt parallel or complementary specimen triage. For example, FFPE tissue or cell blocks may be prioritized for DNA-based mutation profiling, while alcohol-fixed cytology smears, fresh/frozen tissue, exfoliative fluids, or cytology supernatants are preserved for RNA-seq. This approach is particularly relevant in tumor types (e.g., non-small-cell lung carcinoma) where both mutation detection and fusion analysis are essential for comprehensive molecular characterization [158].

Implementation of best practices must also account for cost and accessibility across settings. While approaches such as VPLN, specialized extraction kits, and digital pathology and AI infrastructure can improve performance, many high-impact practices are low cost, instead relying upon affordable yet scalable processes, such as standardized protocols and documentation, optimized thresholds, temperature control during transport, and intentional triage for specimens destined for RNA analysis when feasible. A resource-aware framework for clinical workflows can improve the equity of access to and the accuracy of data generated from meaningful RNA-based testing in a variety of settings regardless of their constraints.

From an operational perspective, laboratories may further enhance efficiency and accessibility by implementing tiered testing algorithms that balance in-house testing with referral to specialized laboratories. Common high-prevalence biomarkers can be tested locally using validated assays, while rare, complex, or low-volume tests are referred to external laboratories with specialized expertise. This hybrid model allows laboratories, particularly those in resource-limited settings, to control costs and turn-around times while maintaining broad molecular testing capabilities.

## Conclusions

RNA sequencing from small biopsies and cytological specimens demands rigorous pre-analytical control to ensure nucleic acid integrity and reproducibility of transcriptomic data. This review highlights the multifactorial influence of ischemia time, fixation chemistry, storage parameters, and preservation methods on RNA yield, fragmentation, and sequencing performance. With the evolution of sequencing platforms that require high-quality input from ever-smaller biospecimens, the development of and adherence to evidence-based, specimen-specific standard operating procedures are essential to maximize analytical validity and data harmonization across clinical and research settings.

Despite ongoing advances in RNA-seq technologies, several limitations remain in the pre-analytical preparation of small biopsies and cytological specimens. Challenges include variable RNA yield and integrity due to factors such as limited cellularity, specimen heterogeneity, and pre-analytical influences such as cold ischemia time, fixation chemistry, and storage conditions. Standard RNA quality metrics, such as RIN and DV200, may not reliably predict sequencing performance in low-input or fragmented samples, particularly those derived from formalin- or alcohol-fixed preparations. Furthermore, most existing protocols have been optimized for fresh or bulk tissue samples and are not directly transferable to minimally invasive specimens. There is a lack of universally accepted, specimen-specific standard operating procedures for cytology smears, liquid-based cytology, and exfoliative fluids, which limits reproducibility and data harmonization across institutions.

Future research should prioritize the development of extraction and library preparation methods tailored to low-input and partially degraded RNA, and the identification of specimen-type-specific quality control thresholds that better predict successful sequencing outcomes. Additionally, systematic studies are needed to evaluate the impact of non-crosslinking or alcohol-based fixation methods on transcriptomic fidelity. Collaborative multi-institutional efforts to establish consensus guidelines for pre-analytical variables across diverse specimen types will be critical for expanding the clinical utility of RNA-seq in precision diagnostics.

## Declarations

### Availability of data and material

Data sharing is not applicable to this article as no datasets were generated or analyzed during the current study.

## References

[1] National Institutes of Health. AR-25-325: integrating biospecimen science approaches into clinical assay development (U01 clinical trial not allowed). 2025 March 31. https://grants.nih.gov/grants/guide/pa-files/PAR-25-325.html. Accessed 2 Oct 2025.

[2] National Institutes of Health. Projects: NIH RePORTER. 2025. https://reporter.nih.gov/search/ek7rJW3pwkSeVhrUQfGZTw/projects. Accessed 2 Oct 2025.

[3] Youn M, Smith SM, Lee AG, et al. Comparison of the Transcriptomic Signatures in Pediatric and Adult CML. Cancers (Basel). 2021;13(24):6263. 10.3390/cancers13246263 PMC8699058 34944883

[4] Rey F, Messa L, Pandini C, et al. RNA-seq characterization of sex-differences in adipose tissue of obesity affected patients: computational analysis of differentially expressed coding and non-coding RNAs. J Pers Med. 2021;11(5):352. 10.3390/jpm11050352 PMC8145808 33924951

[5] Peymani F, Farzeen A, Prokisch H. RNA sequencing role and application in clinical diagnostic. Pediatr Investig. 2022;6(1):29–35. 35382420 10.1002/ped4.12314 PMC8960934

[6] Nibid L, Sabarese G, Andreotti L, et al. RNA-seq analysis in non-small cell lung cancer: what is the best sample from clinical practice? J Pers Med. 2024;14(8):851. 10.3390/jpm14080851 PMC11355753 39202042

[7] Chang E, Wang Y, Zhu R, et al. General anesthetic action profile on the human prefrontal cortex cells through comprehensive single-cell RNA-seq analysis. Science. 2023;26(4):106534. 10.1016/j.isci.2023.106534 PMC10130912 37123239

[8] Moore HM, Kelly AB, Jewell SD, et al. Biospecimen reporting for improved study quality (BRISQ). J Proteome Res. 2011;10(8):3429–3438. 21574648 10.1021/pr200021n PMC3169291

[9] Betsou F, Chuaqui R, De-Wilde A, et al. Standard PREanalytical Code Version 4.0. Biopreserv Biobank. 2025; 23(4):328-332. 10.1089/bio.2024.0010 PMC12416493 39133809

[10] Roy-Chowdhuri S, Dacic S, Ghofrani M, et al. Collection and handling of thoracic small biopsy and cytology specimens for ancillary studies: guideline from the College of American Pathologists in Collaboration with the American College of Chest Physicians, Association for Molecular Pathology, American Society of Cytopathology, American Thoracic Society, Pulmonary Pathology Society, Papanicolaou Society of Cytopathology, Society of Interventional Radiology, and Society of Thoracic Radiology. Arch Pathol Lab Med. Published online May 13, 2020. 10.5858/arpa.2020-0119-CP. 32401054 10.5858/arpa.2020-0119-CP

[11] Giraldo-Parra L, Ramirez LG, Navas A, et al. Quality parameters for RNA preparations from biopsies of ulcerated human skin. Wellcome Open Res. 2022;7:249. 36879918 10.12688/wellcomeopenres.18052.1 PMC9984735

[12] Diep R, MacDonald M, Cooper R, et al. Biopsy method and needle size on success of next-generation sequencing in NSCLC: a brief report. JTO Clin Res Rep. 2023;4(4):100497. 37090100 10.1016/j.jtocrr.2023.100497 PMC10119792

[13] Yeung J, Fotiadis N, Diamantopoulos A, et al. Next-generation sequencing and image-guided tissue sampling: a primer for interventional radiologists. J Vasc Interv Radiol. 2023;34(8):1291-302.e1. 36977432 10.1016/j.jvir.2023.03.012

[14] Watanabe K, Kohsaka S, Tatsuno K, et al. Analysis of quality metrics in comprehensive cancer genomic profiling using a dual DNA-RNA panel. Pract Lab Med. 2024;39:e00368. 38404525 10.1016/j.plabm.2024.e00368 PMC10883814

[15] Lin Y, Dong ZH, Ye TY, et al. Optimization of FFPE preparation and identification of gene attributes associated with RNA degradation. NAR Genom Bioinform. 2024;6(1):lqae008. 38298182 10.1093/nargab/lqae008 PMC10830353

[16] Hewitt SM, Lewis FA, Cao Y, et al. Tissue handling and specimen preparation in surgical pathology: issues concerning the recovery of nucleic acids from formalin-fixed, paraffin-embedded tissue. Arch Pathol Lab Med. 2008;132(12):1929–1935. 19061293 10.5858/132.12.1929

[17] Compton CC, Robb JA, Anderson MW, et al. Preanalytics and precision pathology: pathology practices to ensure molecular integrity of cancer patient biospecimens for precision medicine. Arch Pathol Lab Med. 2019;143(11):1346–1363. 31329478 10.5858/arpa.2019-0009-SA

[18] Bussolati G, Annaratone L, Maletta F. The pre-analytical phase in surgical pathology. Recent Results Cancer Res. 2015;199:1–13. 25636424 10.1007/978-3-319-13957-9_1

[19] Bass BP, Engel KB, Greytak SR, et al. A review of preanalytical factors affecting molecular, protein, and morphological analysis of formalin-fixed, paraffin-embedded (FFPE) tissue: how well do you know your FFPE specimen? Arch Pathol Lab Med. 2014;138(11):1520–1530. 25357115 10.5858/arpa.2013-0691-RA

[20] Gupta S, Chen T, Destenaves B. Quantitative RNA assessment and long-term stability in the FFPE tumor samples using digital spatial profiler. Immunooncol Technol. 2022;13:100069. 35754852 10.1016/j.iotech.2021.100069 PMC9216648

[21] Schmeller J, Wessolly M, Mairinger E, et al. Setting out the frame conditions for feasible use of FFPE derived RNA. Pathol Res Pract. 2019;215(2):381–386. 30606660 10.1016/j.prp.2018.12.027

[22] Schmitt F, Yang Z, Esebua M. Molecular testing in cytology. Diagn Cytopathol. 2023;51(1):3–4. 36367273 10.1002/dc.25071

[23] Vigliar E, Lozano MD, Roy-Chowdhuri S. Editorial: advances in molecular cytopathology. Front Med (Lausanne). 2022;9:851949. 35223934 10.3389/fmed.2022.851949 PMC8873172

[24] Roy-Chowdhuri S. Advances in molecular testing techniques in cytologic specimens. Surg Pathol Clin. 2018;11(3):669–677. 30190147 10.1016/j.path.2018.04.007

[25] Gan Q, Roy-Chowdhuri S. Specimen considerations in molecular oncology testing. Clin Lab Med. 2022;42(3):367–383. 36150817 10.1016/j.cll.2022.04.002

[26] Kasraeian S, Allison DC, Ahlmann ER, et al. A comparison of fine-needle aspiration, core biopsy, and surgical biopsy in the diagnosis of extremity soft tissue masses. Clin Orthop Relat Res. 2010;468(11):2992–3002. 20512437 10.1007/s11999-010-1401-x PMC2947686

[27] Kanagal-Shamanna R, Portier BP, Singh RR, et al. Next-generation sequencing-based multi-gene mutation profiling of solid tumors using fine needle aspiration samples: promises and challenges for routine clinical diagnostics. Mod Pathol. 2014;27(2):314–327. 23907151 10.1038/modpathol.2013.122

[28] Chen H, Luthra R, Goswami RS, et al. Analysis of pre-analytic factors affecting the success of clinical next-generation sequencing of solid organ malignancies. Cancers (Basel). 2015;7(3):1699–1715. 26343728 10.3390/cancers7030859 PMC4586792

[29] Faber E, Grosu H, Sabir S, et al. Adequacy of small biopsy and cytology specimens for comprehensive genomic profiling of patients with non-small-cell lung cancer to determine eligibility for immune checkpoint inhibitor and targeted therapy. J Clin Pathol. 2022;75(9):612–619. 33952592 10.1136/jclinpath-2021-207597

[30] Roy-Chowdhuri S, Chen H, Singh RR, et al. Concurrent fine needle aspirations and core needle biopsies: a comparative study of substrates for next-generation sequencing in solid organ malignancies. Mod Pathol. 2017;30(4):499–508. 28084342 10.1038/modpathol.2016.228

[31] Smouse JH, Cibas ES, Janne PA, et al. EGFR mutations are detected comparably in cytologic and surgical pathology specimens of nonsmall cell lung cancer. Cancer. 2009;117(1):67–72. 19347832 10.1002/cncy.20011

[32] Vigliar E, Malapelle U, De Luca C, et al. Challenges and opportunities of next-generation sequencing: a cytopathologist’s perspective. Cytopathology. 2015;26(5):271–283. 26399861 10.1111/cyt.12265

[33] Kaya C, Dorsaint P, Mercurio S, et al. Limitations of detecting genetic variants from the RNA sequencing data in tissue and fine-needle aspiration samples. Thyroid. 2021;31(4):589–595. 32948110 10.1089/thy.2020.0307 PMC8195874

[34] Marczyk M, Fu C, Lau R, et al. The impact of RNA extraction method on accurate RNA sequencing from formalin-fixed paraffin-embedded tissues. BMC Cancer. 2019;19(1):1189. 31805884 10.1186/s12885-019-6363-0 PMC6896723

[35] Marczyk M, Fu C, Lau R, et al. Assessment of stained direct cytology smears of breast cancer for whole transcriptome and targeted messenger RNA sequencing. Cancer Cytopathol. 2023;131(5):289–99. 36650408 10.1002/cncy.22679 PMC10614161

[36] Sorber L, Claes B, Zwaenepoel K, et al. Evaluation of cytologic sample preparations for compatibility with nucleic acid analysis. Am J Clin Pathol. 2022;157(2):293–304. 34542583 10.1093/ajcp/aqab121 PMC8824667

[37] Sura GH, Tran K, Trevarton AJ, et al. Comparative analysis of Ficoll-Hypaque and CytoLyt techniques for blood removal in breast cancer malignant effusions: effects on RNA quality and sequencing outcomes. J Am Soc Cytopathol. 2025;14(2):91–101. 39668068 10.1016/j.jasc.2024.11.001 PMC12938591

[38] Sura GH, et al. Pre-analytical effects on whole transcriptome and targeted RNA sequencing analysis in cytology: the effects of prolonged time in storage of effusion specimens prior to preservation. Cytopathology. 2023;34(6):551–561. 37712171 10.1111/cyt.13304 PMC10592006

[39] Sura GH, Tran K, Fu C, et al. Molecular testing opportunities on cytology effusion specimens: the pre-analytic effects of various body fluid cytology preparation methods on RNA extraction quality and targeted sequencing. J Am Soc Cytopathol. 2023;12(1):10–19. 36270909 10.1016/j.jasc.2022.09.003 PMC10644714

[40] Engels M, Michael C, Dobra K, et al. Management of cytological material, pre-analytical procedures and bio-banking in effusion cytopathology. Cytopathology. 2019;30(1):31–8. 30430668 10.1111/cyt.12654

[41] Maher MH, Liu D, Flandrin P, et al. A rapid turnaround time workflow for a cytological liquid biopsy assay using FNA supernatant specimens. Cancer Cytopathol. 2025;133(1):e22925. 39704279 10.1002/cncy.22925

[42] Li G, Liu D, Flandrin P, et al. Tumor-derived exosomal RNA from fine-needle aspiration supernatant as a novel liquid biopsy for molecular diagnosis of cancer. Pathol Oncol Res. 2022;28:1610344. 35991837 10.3389/pore.2022.1610344 PMC9388727

[43] Maher MH, Treekitkarnmongkol W, Ghatak S, et al. An integrated multi-omics biomarker approach using molecular profiling and microRNAs for evaluation of pancreatic cyst fluid. Cancer Cytopathol. 2025;133(4):e70008. 40106268 10.1002/cncy.70008 PMC13493043

[44] Kim D, Vanderbilt C, Yang SR, et al. Maximizing the clinical utility and performance of cytology samples for comprehensive genetic profiling: a report on the impact of process optimization through the analysis of 4871 cytology samples profiled by MSK-IMPACT. Nat Commun. 2025;16(1):116. 10.1038/s41467-024-55456-8 PMC11696557 39747849

[45] Roy-Chowdhuri S. The bridge: Sspernatant derived from cytological sample preparations. Cytopathology. 2025;36(3):222–227. 39921463 10.1111/cyt.13475

[46] Wang X, Li S, Ou R, et al. Wide-spectrum profiling of plasma cell-free RNA and the potential for health-monitoring. RNA Biol. 2025;22(1):1–15. 40110666 10.1080/15476286.2025.2481736 PMC11970758

[47] Zhong P, Bai L, Hong M, et al. A comprehensive review on circulating cfRNA in plasma: implications for disease diagnosis and beyond. Diagnostics (Basel). 2024;14(10):1045. 10.3390/diagnostics14101045. 38786343 10.3390/diagnostics14101045 PMC11119755

[48] Liu T, Li Z, Chen S, et al. Circulating cell-free DNA fragmentomics detection and beyond. Aging Dis. 2025. 10.14336/AD.2025.1029 PMC13437109 40956966

[49] Tsui WHA, Jiang P, Lo YMD. Cell-free DNA fragmentomics in cancer. Cancer Cell. 2025;43(10):1792–1814. 41043439 10.1016/j.ccell.2025.09.006

[50] Sun Y, Yu H, Han S, et al. Method for the extraction of circulating nucleic acids based on MOF reveals cell-free RNA signatures in liver cancer. Natl Sci Rev. 2024;11(1):nwae022. 38348130 10.1093/nsr/nwae022 PMC10860518

[51] Carithers LJ, Agarwal R, Guan P, et al. The biospecimen preanalytical variables program: a multiassay comparison of effects of delay to fixation and fixation duration on nucleic acid quality. Arch Pathol Lab Med. 2019;143(9):1106–1118. 30785788 10.5858/arpa.2018-0172-OA

[52] van der Wijngaart H, Jagga S, Dekker H, et al. Advancing wide implementation of precision oncology: a liquid nitrogen-free snap freezer preserves molecular profiles of biological samples. Cancer Med. 2023;12(9):10979–10989. 36916528 10.1002/cam4.5781 PMC10225239

[53] Servais MD, Galtier F, Nouvel A, et al. Addressing the quality challenge of a human biospecimen biobank through the creation of a quality management system. PLoS One. 2022;17(12):e0278780. 36584180 10.1371/journal.pone.0278780 PMC9803146

[54] Madissoon E, Wilbrey-Clark A, Miragaia RJ, et al. scRNA-seq assessment of the human lung, spleen, and esophagus tissue stability after cold preservation. Genome Biol. 2019;21(1):1. 31892341 10.1186/s13059-019-1906-x PMC6937944

[55] Jones W, Greytak S, Odeh H, et al. Deleterious effects of formalin-fixation and delays to fixation on RNA and miRNA-Seq profiles. Sci Rep. 2019;9(1):6980. 31061401 10.1038/s41598-019-43282-8 PMC6502812

[56] Mathieson W, Mommaerts K, Trouet JM, et al. Cold ischemia score: an mRNA assay for the detection of extended cold ischemia in formalin-fixed, paraffin-embedded tissue. J Histochem Cytochem. 2019;67(3):159–168. 30562131 10.1369/0022155418819967 PMC6393842

[57] Zhang X, Han QY, Zhao ZS, et al. Biobanking of fresh-frozen gastric cancer tissues: impact of long-term storage and clinicopathological variables on RNA quality. Biopreserv Biobank. 2019;17(1):58–63. 30457887 10.1089/bio.2018.0038

[58] Pedersen IS, Thomassen M, Tan Q, et al. Differential effect of surgical manipulation on gene expression in normal breast tissue and breast tumor tissue. Mol Med. 2018;24(1):57. 30445902 10.1186/s10020-018-0058-x PMC6240321

[59] Castells Domingo X, Ferrer-Font L, Davila M, et al. Improving ribosomal RNA integrity in surgically resected human brain tumor biopsies. Biopreserv Biobank. 2016;14(2):156–164. 26886080 10.1089/bio.2015.0086

[60] Olsen J, Kirkeby LT, Eiholm S, et al. Impact of in vivo ischemic time on RNA quality: experiences from a colon cancer biobank. Biopreserv Biobank. 2015;13(4):255–262. 26186671 10.1089/bio.2015.0009

[61] Sun H, Sun R, Hao M, et al. Effect of duration of ex vivo ischemia time and storage period on RNA quality in biobanked human renal cell carcinoma tissue. Ann Surg Oncol. 2016;23(1):297–304. 25567356 10.1245/s10434-014-4327-9

[62] David KA, Unger FT, Uhlig P, et al. Surgical procedures and postsurgical tissue processing significantly affect expression of genes and EGFR-pathway proteins in colorectal cancer tissue. Oncotarget. 2014;5(22):11017–11028. 25526028 10.18632/oncotarget.2669 PMC4294341

[63] Aktas B, Sun H, Yao H, et al. Global gene expression changes induced by prolonged cold ischemic stress and preservation method of breast cancer tissue. Mol Oncol. 2014;8(3):717–727. 24602449 10.1016/j.molonc.2014.02.002 PMC4048748

[64] Musella V, Verderio P, Reid JF, et al. Effects of warm ischemic time on gene expression profiling in colorectal cancer tissues and normal mucosa. PLoS One. 2013;8(1):e53406. 23308215 10.1371/journal.pone.0053406 PMC3538764

[65] Liu NW, Sanford T, Srinivasan R, et al. Impact of ischemia and procurement conditions on gene expression in renal cell carcinoma. Clin Cancer Res. 2013;19(1):42–49. 23136194 10.1158/1078-0432.CCR-12-2606 PMC3658320

[66] Bao WG, Zhang X, Zhang JG, et al. Biobanking of fresh-frozen human colon tissues: impact of tissue ex-vivo ischemia times and storage periods on RNA quality. Ann Surg Oncol. 2013;20(5):1737–1744. 10.1245/s10434-012-2440-1 22711177

[67] Hatzis C, Sun H, Yao H, et al. Effects of tissue handling on RNA integrity and microarray measurements from resected breast cancers. J Natl Cancer Inst. 2011;103(24):1871–1883. 10.1093/jnci/djr438 PMC3243675 22034635

[68] Borgan E, Navon R, Vollan HK, et al. Ischemia caused by time to freezing induces systematic microRNA and mRNA responses in cancer tissue. Mol Oncol. 2011;5(6):564–576. 21917534 10.1016/j.molonc.2011.08.004 PMC5528325

[69] Hůlková M, Zeman J. Placental tissue as model for pilot study focused on RNA analysis from human foetal tissue. Prague Med Rep. 2011;112(2):93–101. 21699758

[70] Symmans WF, Hatzis C, Sotiriou C, et al. Genomic index of sensitivity to endocrine therapy for breast cancer. J Clin Oncol. 2010;28(27):4111–4119. 20697068 10.1200/JCO.2010.28.4273 PMC2953969

[71] Rudloff U, Bhanot U, Gerald W, et al. Biobanking of human pancreas cancer tissue: impact of ex-vivo procurement times on RNA quality. Ann Surg Oncol. 2010;17(8):2229–2236. 20162455 10.1245/s10434-010-0959-6 PMC3877690

[72] Bray SE, Paulin FE, Fong SC, et al. Gene expression in colorectal neoplasia: modifications induced by tissue ischaemic time and tissue handling protocol. Histopathology. 2010;56(2):240–250. 20102403 10.1111/j.1365-2559.2009.03470.x

[73] De Cecco L, Musella V, Veneroni S, et al. Impact of biospecimens handling on biomarker research in breast cancer. BMC Cancer. 2009;9:409. 19930681 10.1186/1471-2407-9-409 PMC2787533

[74] Chung JY, Braunschweig T, Williams R, et al. Factors in tissue handling and processing that impact RNA obtained from formalin-fixed, paraffin-embedded tissue. J Histochem Cytochem. 2008;56(11):1033–1042. 18711211 10.1369/jhc.2008.951863 PMC2569903

[75] van Maldegem F, de Wit M, Morsink F, et al. Effects of processing delay, formalin fixation, and immunohistochemistry on RNA recovery from formalin-fixed paraffin-embedded tissue sections. Diagn Mol Pathol. 2008;17(1):51–58. 18303406 10.1097/PDM.0b013e31814b8866

[76] Jewell SD, Srinivasan M, McCart LM, et al. Analysis of the molecular quality of human tissues: an experience from the Cooperative Human Tissue Network. Am J Clin Pathol. 2002;118(5):733–741. 12428794 10.1309/VPQL-RT21-X7YH-XDXK

[77] Guerrera F, Tabbò F, Bessone L, et al. The influence of tissue ischemia time on RNA integrity and patient-derived xenografts (PDX) engraftment rate in a non-small cell lung cancer (NSCLC) niobank. PLoS One. 2016;11(1):e0145100. 26731692 10.1371/journal.pone.0145100 PMC4701130

[78] Fan XJ, Huang Y, Wu PH, et al. Impact of cold ischemic time and freeze-thaw cycles on RNA, DNA and protein quality in colorectal cancer tissues biobanking. J Cancer. 2019;10(20):4978–4988. 31598170 10.7150/jca.29372 PMC6775519

[79] Srinivasan M, Sedmak D, Jewell S. Effect of fixatives and tissue processing on the content and integrity of nucleic acids. Am J Pathol. 2002;161(6):1961–1971. 12466110 10.1016/S0002-9440(10)64472-0 PMC1850907

[80] Agrawal L, Engel KB, Greytak SR, et al. Understanding preanalytical variables and their effects on clinical biomarkers of oncology and immunotherapy. Semin Cancer Biol. 2018;52(Pt 2):26–38. 29258857 10.1016/j.semcancer.2017.12.008 PMC6004232

[81] Grizzle WE, Otali D, Sexton KC, et al. Effects of cold ischemia on gene expression: a review and commentary. Biopreserv Biobank. 2016;14(6):548–558. 27551929 10.1089/bio.2016.0013 PMC5180081

[82] Somiari SB, Shuss S, Liu J, et al. Assessing the quality of RNA isolated from human breast tissue after ambient room temperature exposure. PloS ONE. 2022;17(1):e0262654. 35041696 10.1371/journal.pone.0262654 PMC8765617

[83] Freidin MB, Bhudia N, Lim E, et al. Impact of collection and storage of lung tumor tissue on whole genome expression profiling. J Mol Diagn. 2012;14(2):140–148. 22240448 10.1016/j.jmoldx.2011.11.002 PMC3547171

[84] Dietel M, Bubendorf L, Dingemans AM, et al. Diagnostic procedures for non-small-cell lung cancer (NSCLC): recommendations of the European Expert Group. Thorax. 2016;71(2):177–184. 26530085 10.1136/thoraxjnl-2014-206677 PMC4752623

[85] Michael CW, Davidson B. Pre-analytical issues in effusion cytology. Pleura Peritoneum. 2016;1(1):45–56. 30911607 10.1515/pp-2016-0001 PMC6328068

[86] Wolff AC, Hammond MEH, Allison KH, et al. Human epidermal growth factor receptor 2 testing in breast cancer: American Society of Clinical Oncology/College of American Pathologists clinical practice guideline focused update. J Clin Oncol. 2018;36(20):2105–2122. 29846122 10.1200/JCO.2018.77.8738

[87] Antonangelo L, Vargas FS, Acencio MM, et al. Effect of temperature and storage time on cellular analysis of fresh pleural fluid samples. Cytopathology. 2012;23(2):103–107. 21418346 10.1111/j.1365-2303.2011.00863.x

[88] Manosca F, Schinstine M, Fetsch PA, et al. Diagnostic effects of prolonged storage on fresh effusion samples. Diagn Cytopathol. 2007;35(1):6–11. 17173298 10.1002/dc.20587

[89] Suntsova M, Gaifullin N, Allina D, et al. Atlas of RNA sequencing profiles for normal human tissues. Sci Data. 2019;6(1):36. 31015567 10.1038/s41597-019-0043-4 PMC6478850

[90] Eikrem O, Beisland C, Hjelle K, et al. Transcriptome sequencing (RNAseq) enables utilization of formalin-fixed, paraffin-embedded biopsies with clear cell renal cell carcinoma for exploration of disease biology and biomarker development. PLoS ONE. 2016;11(2):e0149743. 26901863 10.1371/journal.pone.0149743 PMC4764764

[91] Thavarajah R, Mudimbaimannar VK, Elizabeth J, et al. Chemical and physical basics of routine formaldehyde fixation. J Oral Maxillofac Pathol. 2012;16(3):400–405. 23248474 10.4103/0973-029X.102496 PMC3519217

[92] Howat WJ, Wilson BA. Tissue fixation and the effect of molecular fixatives on downstream staining procedures. Methods. 2014;70(1):12–19. 24561827 10.1016/j.ymeth.2014.01.022 PMC4240801

[93] Sorokin M, Lyadov V, Suntsova M, et al. Detection of fusion events by RNA sequencing in FFPE versus freshly frozen colorectal cancer tissue samples. Front Mol Biosci. 2024;11:1448792. 39906487 10.3389/fmolb.2024.1448792 PMC11791353

[94] Jacobsen SB, Tfelt-Hansen J, Smerup MH, et al. Comparison of whole transcriptome sequencing of fresh, frozen, and formalin-fixed, paraffin-embedded cardiac tissue. PLoS ONE. 2023;18(3):e0283159. 36989279 10.1371/journal.pone.0283159 PMC10058139

[95] Skaftason A, Qu Y, Abdulla M, et al. Transcriptome sequencing of archived lymphoma specimens is feasible and clinically relevant using exome capture technology. Genes Chromosomes Cancer. 2022;61(1):27–36. 34647650 10.1002/gcc.23002

[96] Boneva S, Schlecht A, Böhringer D, et al. 3’ MACE RNA-sequencing allows for transcriptome profiling in human tissue samples after long-term storage. Lab Investig. 2020;100(10):1345–1355. 32467590 10.1038/s41374-020-0446-z PMC7498368

[97] Esteva-Socias M, Gómez-Romano F, Carrillo-Ávila JA, et al. Impact of different stabilization methods on RT-qPCR results using human lung tissue samples. Sci Rep. 2020;10(1):3579. 32108147 10.1038/s41598-020-60618-x PMC7046779

[98] Li J, Fu C, Speed TP, et al. Accurate RNA sequencing from formalin fixed cancer tissue to represent high-quality transcriptome from frozen tissue. JCO Precis Oncol. 2018;2018. 10.1200/PO.17.00091 PMC5976456 29862382

[99] Li P, Conley A, Zhang H, et al. Whole-transcriptome profiling of formalin-fixed, paraffin-embedded renal cell carcinoma by RNA-seq. BMC Genom. 2014;15(1):1087. 10.1186/1471-2164-15-1087 PMC4298956 25495041

[100] Hedegaard J, Thorsen K, Lund MK, et al. Next-generation sequencing of RNA and DNA isolated from paired fresh-frozen and formalin-fixed paraffin-embedded samples of human cancer and normal tissue. PLoS One. 2014;9(5):e98187. 24878701 10.1371/journal.pone.0098187 PMC4039489

[101] Norton N, Sun Z, Asmann YW, et al. Gene expression, single nucleotide variant and fusion transcript discovery in archival material from breast tumors. PLoS One. 2013;8(11):e81925. 24278466 10.1371/journal.pone.0081925 PMC3838386

[102] Meng W, McElroy JP, Volinia S, et al. Comparison of microRNA deep sequencing of matched formalin-fixed paraffin-embedded and fresh frozen cancer tissues. PLoS One. 2013;8(5):e64393. 23696889 10.1371/journal.pone.0064393 PMC3655971

[103] Groelz D, Sobin L, Branton P, et al. Non-formalin fixative versus formalin-fixed tissue: a comparison of histology and RNA quality. Exp Mol Pathol. 2013;94(1):188–194. 22814231 10.1016/j.yexmp.2012.07.002

[104] Turashvili G, Yang W, McKinney S, et al. Nucleic acid quantity and quality from paraffin blocks: defining optimal fixation, processing and DNA/RNA extraction techniques. Exp Mol Pathol. 2012;92(1):33–43. 21963600 10.1016/j.yexmp.2011.09.013

[105] Weng L, Wu X, Gao H, et al. MicroRNA profiling of clear cell renal cell carcinoma by whole-genome small RNA deep sequencing of paired frozen and formalin-fixed, paraffin-embedded tissue specimens. J Pathol. 2010;222(1):41–51. 20593407 10.1002/path.2736

[106] Sunagawa Y, Hayashi M, Yamada S, et al. Impact of molecular surgical margin analysis on the prediction of pancreatic cancer recurrences after pancreaticoduodenectomy. Clin Epigenet. 2021;13(1):172. 10.1186/s13148-021-01165-8 PMC8444591 34530906

[107] Gaston SM, Soares MA, Siddiqui MM, et al. Tissue-print and print-phoresis as platform technologies for the molecular analysis of human surgical specimens: mapping tumor invasion of the prostate capsule. Nat Med. 2005;11(1):95–101. 15619629 10.1038/nm1169

[108] Wang F, Wang L, Briggs C, et al. DNA degradation test predicts success in whole-genome amplification from diverse clinical samples. J Mol Diagn. 2007;9(4):441–451. 17690213 10.2353/jmoldx.2007.070004 PMC1975106

[109] Roh JL, Westra WH, Califano JA, et al. Tissue imprint for molecular mapping of deep surgical margins in patients with head and neck squamous cell carcinoma. Head Neck. 2012;34(11):1529–1536. 22223471 10.1002/hed.21982 PMC3377010

[110] Van Neste L, Groskopf J, Grizzle WE, et al. Epigenetic risk score improves prostate cancer risk assessment. Prostate. 2017;77(12):1259–64. 28762545 10.1002/pros.23385

[111] Engel KB, Vaught J, Moore HM. National Cancer Institute biospecimen evidence-based practices: a novel approach to pre-analytical standardization. Biopreserv Biobank. 2014;12(2):148–150. 24749882 10.1089/bio.2013.0091 PMC3995433

[112] Auer H, Mobley JA, Ayers LW, et al. The effects of frozen tissue storage conditions on the integrity of RNA and protein. Biotech Histochem. 2014;89(7):518–528. 24799092 10.3109/10520295.2014.904927 PMC4213858

[113] Steu S, Baucamp M, von Dach G, et al. A procedure for tissue freezing and processing applicable to both intra-operative frozen section diagnosis and tissue banking in surgical pathology. Virchows Arch. 2008;452(3):305–312. 18253747 10.1007/s00428-008-0584-y

[114] Fang Y, Peng Z, Wang Y, et al. Improvements and challenges of tissue preparation for spatial transcriptome analysis of skull base tumors. Heliyon. 2023;9(3):e14133. 36938455 10.1016/j.heliyon.2023.e14133 PMC10018477

[115] Gaston SM, Grizzle WE, Rais-Bahrami S, et al. Nitrocellulose tissue prints: an innovative approach to preparing high quality DNA and RNA from prostate biopsies without compromising the cores for pathology diagnosis. Transl Androl Urol. 2018;7(Suppl. 4):S514–S518. 10.21037/tau.2018.09.01 PMC6178309 30363516

[116] Ramani NS, Chen H, Broaddus RR, et al. Utilization of cytology smears improves success rates of RNA-based next-generation sequencing gene fusion assays for clinically relevant predictive biomarkers. Cancer Cytopathol. 2021;129(5):374–382. 10.1002/cncy.22381 PMC12002355 33119213

[117] Kriukov K, Ivchenkov D, Bejanyan A, et al. Morphological bone score as a predictive tool for molecular profiling success. J Mol Diagn. 2025;27(8):747–756. 10.1016/j.jmoldx.2025.04.013 40517895

[118] Miquelestorena-Standley E, Jourdan ML, Collin C, et al. Effect of decalcification protocols on immunohistochemistry and molecular analyses of bone samples. Mod Pathol. 2020;33(8):1505–1517. 32094425 10.1038/s41379-020-0503-6

[119] van Limbeek MAJ, Jagga S, Holland H, et al. Cooling of a vial in a snapfreezing device without using sacrificial cryogens. Sci Rep. 2019;9(1):3510. 10.1038/s41598-019-40115-6 PMC6400931 30837583

[120] Hubel A, Spindler R, Skubitz AP. Storage of human biospecimens: selection of the optimal storage temperature. Biopreserv Biobank. 2014;12(3):165–175. 24918763 10.1089/bio.2013.0084

[121] Biskup E, Schejbel L, de Oliveira DNP, et al. Test of the FlashFREEZE unit in tissue samples freezing for biobanking purposes. Cell Tissue Bank. 2023;24(2):435–447. 36309911 10.1007/s10561-022-10045-1 PMC10209260

[122] Sanchez-Carbayo M, Saint F, Lozano JJ, et al. Comparison of gene expression profiles in laser-microdissected, nonembedded, and OCT-embedded tumor samples by oligonucleotide microarray analysis. Clin Chem. 2003;49(12):2096–2100. 14633888 10.1373/clinchem.2003.017525

[123] Xu J, Pandoh PK, Corbett RD, et al. A high-throughput pipeline for DNA/RNA/small RNA purification from tissue samples for sequencing. Biotechniques. 2023;75(2):47–55. 37551834 10.2144/btn-2023-0011

[124] Hentze JL, Kringelbach TM, Novotny GW, et al. Optimized biobanking procedures for preservation of RNA in tissue: comparison of snap-freezing and RNAlater-fixation methods. Biopreserv Biobank. 2019;17(6):562–569. 31618057 10.1089/bio.2019.0028

[125] Hatanaka KC, Nakamura K, Katoh R, et al. Impact of the quality of resected thyroid cancer tissue sample on next-generation sequencing testing. Pathol Int. 2024;74(2):77–86. 38226479 10.1111/pin.13399 PMC11551834

[126] Amemiya K, Hirotsu Y, Nagakubo Y, et al. Influence of formalin fixation duration on RNA quality and quantity from formalin-fixed paraffin-embedded hepatocellular carcinoma tissues. Pathol Int. 2023;73(12):593–600. 37933792 10.1111/pin.13385

[127] Iorgulescu JB, Yang, RK, Roy-Chowdhuri S, et al. Same-day molecular testing for targetable mutations in solid tumor cytopathology: the next frontier of the rapid on-site evaluation. Cancer Cytopathol. 2025;133(1):e22930. 39746874 10.1002/cncy.22930

[128] Wang T, Qian X, Wang Z, et al. Detection of cell-free *BIRC5* mRNA in effusions and its potential diagnostic value for differentiating malignant and benign effusions. Int J Cancer. 2009;125(8):1921–1925. 19585499 10.1002/ijc.24516

[129] Roy-Chowdhuri S, Chow CW, Kane MK, et al. Optimizing the DNA yield for molecular analysis from cytologic preparations. Cancer Cytopathol. 2016;124(4):254–260. 26630358 10.1002/cncy.21664

[130] Zhou W, Geiersbach K, Chadwick B. Rapid removal of cytology slide coverslips for DNA and RNA isolation. J Am Soc Cytopathol. 2017;6(1):24–27. 31042630 10.1016/j.jasc.2016.08.005

[131] Sabarinath B, Protyusha GB, Sivapathasundharam B, et al. Role of dry ice in decoverslipping of microscopic slides: a new insight. J Oral Maxillofac Pathol. 2023;27(3):598. 38033942 10.4103/jomfp.jomfp_332_22 PMC10683887

[132] Angelucci A, Pace G, Sanita P, et al. Tissue print of prostate biopsy: a novel tool in the diagnostic procedure of prostate cancer. Diagn Pathol. 2011;6:34. 21489246 10.1186/1746-1596-6-34 PMC3086855

[133] Kofanova O, Paul S, Pexaras A, et al. Biospecimen qualification in a clinical biobank of urological diseases. Biopreserv Biobank. 2024;22(3):257–267. 37878356 10.1089/bio.2022.0190

[134] Vaksman O, Trope C, Davidson B, et al. Exosome-derived miRNAs and ovarian carcinoma progression. Carcinogenesis. 2014;35(9):2113–2120. 24925027 10.1093/carcin/bgu130

[135] Xie L, Chen X, Wang L, et al. Cell-free miRNAs may indicate diagnosis and docetaxel sensitivity of tumor cells in malignant effusions. BMC Cancer. 2010;10:591. 21029414 10.1186/1471-2407-10-591 PMC2988750

[136] Patel NM, Jo H, Eberhard DA, et al. Improved tumor purity metrics in next-generation sequencing for clinical practice: the Integrated Interpretation of Neoplastic Cellularity and Sequencing Results (IINCaSe) approach. Appl Immunohistochem Mol Morphol. 2019;27(10):764–772. 30102605 10.1097/PAI.0000000000000684 PMC6887630

[137] Gertych A, Zurek N Piaseczna N, et al. Tumor cellularity assessment using artificial intelligence trained on immunohistochemistry-restained slides improves selection of lung adenocarcinoma samples for molecular testing. Am J Pathol. 2025;195(5):907–922. 10.1016/j.ajpath.2025.01.009 39892778

[138] L’Imperio V, Cazzaniga G, Mannino M, et al. Digital counting of tissue cells for molecular analysis: the QuANTUM pipeline. Virchows Arch. 2025;486(2):277–286. 10.1007/s00428-024-03794-9 PMC11876257 38532196

[139] Jeong J, Kim D, Ryu YM, et al. Artificial intelligence algorithm for neoplastic cell percentage estimation and its application to copy number variation in urinary tract cancer. J Pathol Transl Med. 2024;58(5):229–240. 39112099 10.4132/jptm.2024.07.13 PMC11424195

[140] Cai G, Adeniran AJ, SpringerLink. Rapid On-site Evaluation (ROSE): a practical guide. 1st ed. Cham: Springer International Publishing; 2019: Imprint.

[141] Satturwar S, Rekhtman N, Lin O, et al. An update on touch preparations of small biopsies. J Am Soc Cytopathol. 2020;9(5):322–331. 32417160 10.1016/j.jasc.2020.04.004 PMC8375623

[142] Walsh EM, Halushka MK. A comparison of tissue dissection techniques for diagnostic, prognostic, and theragnostic analysis of human disease. Pathobiology. 2023;90(3):199–208. 35952628 10.1159/000525979 PMC9918608

[143] Stockley TL, Lo B, Box A, et al. Consensus recommendations to optimize the detection and reporting of NTRK gene fusions by RNA-based next-generation sequencing. Curr Oncol. 2023;30(4):3989–3997. 37185415 10.3390/curroncol30040302 PMC10136625

[144] Newton Y, Sedgwick AJ, Cisneros L, et al. Large scale, robust, and accurate whole transcriptome profiling from clinical formalin-fixed paraffin-embedded samples. Sci Rep. 2020;10(1):17597. 33077815 10.1038/s41598-020-74483-1 PMC7572424

[145] Alsagaby SA. Investigating the impact of RNA integrity variation on the transcriptome of human leukemic cells. 3 Biotech. 2022;12(8):160. 35814037 10.1007/s13205-022-03223-1 PMC9259771

[146] Wu W, Lovett JL, Shedden K, et al. Targeted RNA-seq improves efficiency, resolution, and accuracy of allele specific expression for human term placentas. G3 (Bethesda). 2021;11(8):jkab176. 10.1093/g3journal/jkab176 PMC8496276 34009305

[147] Kornienko IV, Aramova OY, Tishchenko AA, et al. RNA stability: a review of the role of structural features and environmental conditions. Molecules. 2024;29(24):5978. 10.3390/molecules29245978. 39770066 10.3390/molecules29245978 PMC11676819

[148] Olivieri EH, Franco Lde A, Pereira RG, et al. Biobanking practice: RNA storage at low concentration affects integrity. Biopreserv Biobank. 2014;12(1):46–52. 24620769 10.1089/bio.2013.0056

[149] CLSI. Collection, transport, preparation, and storage of specimens for molecular methods: CLSI guideline MM13. Wayne (PA): 2nd ed. Clinical and Laboratory Standards Institute; 2020. Pennsylvania, USA.

[150] Imbeaud S. Towards standardization of RNA quality assessment using user-independent classifiers of microcapillary electrophoresis traces. Nucleic Acids Res. 2005;33(6):e56. 15800207 10.1093/nar/gni054 PMC1072807

[151] Schroeder A, Mueller O, Stocker S, et al. The RIN: an RNA integrity number for assigning integrity values to RNA measurements. BMC Mol Biol. 2006;7:3. 16448564 10.1186/1471-2199-7-3 PMC1413964

[152] Lalmahomed ZS, Coebergh van den Braak RRJ, Oomen MHA, et al. Multicenter fresh frozen tissue sampling in colorectal cancer: does the quality meet the standards for state of the art biomarker research? Cell Tissue Bank. 2017;18(3):425–431. 28258397 10.1007/s10561-017-9613-x PMC5587614

[153] Morente MM, Mager R, Alonso S, et al. TuBaFrost 2: standardising tissue collection and quality control procedures for a European virtual frozen tissue bank network. Eur J Cancer. 2006;42(16):2684–2691. 17027255 10.1016/j.ejca.2006.04.029

[154] Matsubara T, Soh J, Morita M, et al. DV200 index for assessing RNA integrity in next-generation sequencing. Biomed Res Int. 2020;2020:9349132. 32185225 10.1155/2020/9349132 PMC7063185

[155] Walker JE, Oliver JC, Stewart AM, et al. Measuring up: a comparison of TapeStation 4200 and Bioanalyzer 2100 as measurement tools for RNA quality in postmortem human brain samples. Int J Mol Sci. 2023;24(18):13795. 10.3390/ijms241813795. 37762097 10.3390/ijms241813795 PMC10531353

[156] Wang L, Nie J, Sicotte, et al. Measure transcript integrity using RNA-seq data. BMC Bioinform. 2016;17:58. 10.1186/s12859-016-0922-z PMC4739097 26842848

[157] Ewalt MD, Hsiao SJ. Molecular methods: clinical utilization and designing a test menu. Surg Pathol Clin. 2021;14(3):359–368. 34373088 10.1016/j.path.2021.05.001

[158] Lindeman NI, Cagle PT, Aisner DL, et al. Updated molecular testing guideline for the selection of lung cancer patients for treatment with targeted tyrosine kinase inhibitors: guideline from the College of American Pathologists, the International Association for the Study of Lung Cancer, and the Association for Molecular Pathology. Arch Pathol Lab Med. 2018;142(3):321–346. 29355391 10.5858/arpa.2017-0388-CP

[159] Nagakubo Y, Hirotsu Y, Amemiya K, et al. Nucleic Acid Quality Assessment is Critical to the Success of theOncomine Dx Target Test for Lung Cancer. Mol Diagn Ther. 2023;27(4):513–523. 10.1007/s40291-023-00653-2. 10.1007/s40291-023-00653-2 37198423

